# CD137 agonism enhances anti-PD1 induced activation of expanded CD8^+^ T cell clones in a neoadjuvant pancreatic cancer clinical trial

**DOI:** 10.1016/j.isci.2024.111569

**Published:** 2024-12-10

**Authors:** Janelle M. Montagne, Jacob T. Mitchell, Joseph A. Tandurella, Eric S. Christenson, Ludmila V. Danilova, Atul Deshpande, Melanie Loth, Dimitrios N. Sidiropoulos, Emily Davis-Marcisak, Daniel R. Bergman, Qingfeng Zhu, Hao Wang, Luciane T. Kagohara, Logan L. Engle, Benjamin F. Green, Alexander V. Favorov, Won Jin Ho, Su Jin Lim, Rui Zhang, Pan Li, Jessica Gai, Guanglan Mo, Sarah Mitchell, Rulin Wang, Ajay Vaghasia, Wenpin Hou, Yao Xu, Jacquelyn W. Zimmerman, Jennifer H. Elisseeff, Srinivasan Yegnasubramanian, Robert A. Anders, Elizabeth M. Jaffee, Lei Zheng, Elana J. Fertig

**Affiliations:** 1 Department of Oncology, Sidney Kimmel Comprehensive Cancer Center, Johns Hopkins University School of Medicine, Baltimore, MD, USA; 2 Convergence Institute, Johns Hopkins University School of Medicine, Baltimore, MD, USA; 3 Bloomberg Kimmel Immunology Institute, Johns Hopkins University School of Medicine, Baltimore, MD, USA; 4 The Skip Viragh Center for Clinical and Translational Research, Johns Hopkins University School of Medicine, Baltimore, MD, USA; 5 Department of Genetic Medicine, Johns Hopkins School of Medicine, Baltimore MD, USA; 6 Department of Pathology, Johns Hopkins University School of Medicine, Baltimore, MD, USA; 7 Department of Dermatology, Johns Hopkins University School of Medicine, Baltimore, MD, USA; 8 Laboratory of Systems Biology and Computational Genetics, Vavilov Institute of General Genetics, Moscow, RF, Russia; 9 Department of Biostatistics, Johns Hopkins University Bloomberg School of Public Health, Baltimore, MD, USA; 10 Translational Tissue Engineering Center, Wilmer Eye Institute, Johns Hopkins University School of Medicine, Baltimore, MD, USA; 11 Department of Biomedical Engineering, Johns Hopkins University School of Medicine, Baltimore, MD, USA; 12 Department of Radiation Oncology, Johns Hopkins University School of Medicine, Baltimore, MD, USA; 13 InHealth Precision Medicine Program, Johns Hopkins University School of Medicine, Baltimore, MD, USA; 14 The Skip Viragh Pancreatic Cancer Center, Johns Hopkins University School of Medicine, Baltimore, MD, USA; 15 The Pancreatic Cancer Precision Medicine Center of Excellence Program, Johns Hopkins University School of Medicine, Baltimore, MD, USA; 16 Department of Surgery, Johns Hopkins University School of Medicine, Baltimore, MD, USA; 17 Department of Applied Mathematics and Statistics, Johns Hopkins University Whiting School of Engineering, Baltimore, MD, USA

**Keywords:** Health sciences, Medicine, Medical specialty, Internal medicine, Oncology

## Abstract

Successful pancreatic ductal adenocarcinoma (PDAC) immunotherapy requires therapeutic combinations that induce quality T cells. Tumor microenvironment (TME) analysis following therapeutic interventions can identify response mechanisms, informing design of effective combinations. We provide a reference single-cell dataset from tumor-infiltrating leukocytes (TILs) from a human neoadjuvant clinical trial comparing the granulocyte-macrophage colony-stimulating factor (GM-CSF)-secreting allogeneic PDAC vaccine GVAX alone, in combination with anti-PD1 or with both anti-PD1 and CD137 agonist. Treatment with GVAX and anti-PD-1 led to increased CD8^+^ T cell activation and expression of cytoskeletal and extracellular matrix (ECM)-interacting components. Addition of CD137 agonist increased abundance of clonally expanded CD8^+^ T cells and increased immunosuppressive TREM2 signaling in tumor associated macrophages (TAMs), identified by comparison of ligand-receptor networks, corresponding to changes in metabolism and ECM interactions. These findings associate therapy with GVAX, anti-PD1, and CD137 agonist with enhanced CD8^+^ T cell function while inducing alternative immunosuppressive pathways in patients with PDAC.

## Highlights

• Allogeneic PDAC vaccine with anti-PD-1 enhances CD8^+^ T cell activation and ECM interaction
• Inclusion of CD137 agonist increases clonal T cell abundance and macrophage TREM2 signaling

## Graphical abstract

**Figure:**
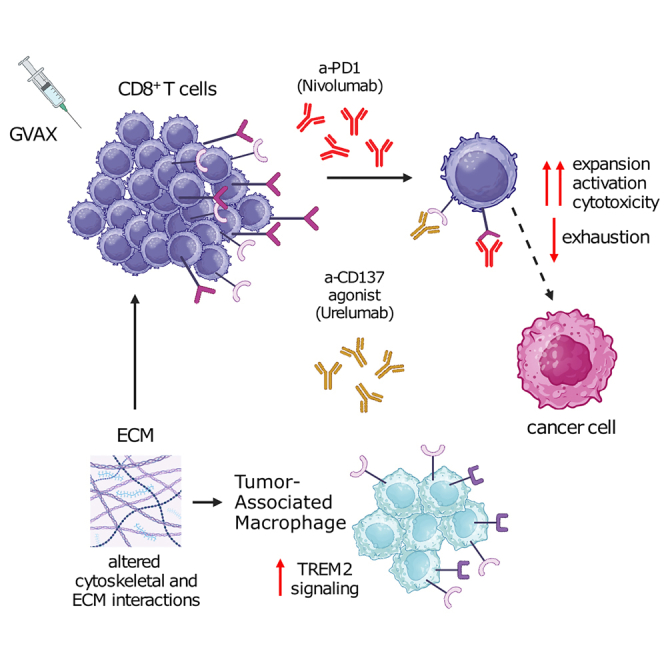

## Introduction

Pancreatic ductal adenocarcinoma (PDAC) continues to rise in incidence and mortality, with an alarming and stagnant 5-year overall survival rate of 11%.^1^ Unlike many cancers, most PDAC patients do not benefit from immune checkpoint inhibitor (ICI) single-agent or combination therapy, including anti-PD1 and anti-CTLA4.^2^ Mechanisms of resistance to ICIs are multifactorial. Most PDAC tumors have a modest mutational burden^3^ and thus few neoantigens for presentation and activation of high-quality effector T cells. Additionally, multiple cellular components contribute to a highly immunosuppressive tumor microenvironment (TME)^4^ and actively promote ICI resistance by preventing TME trafficking, activation, and function of anti-tumor T cells. Thus, combination treatment strategies provide the potential to circumvent these challenges and promote productive anti-tumor immune responses in PDACs.^4^ Although informed by strong preclinical studies, immunotherapy has had limited success in PDAC clinical trials.^5^ Deciphering the impact of treatments on individual cell populations directly isolated from the human PDAC TME is therefore essential to infer the cellular mechanisms of response and resistance and inform the design of more effective combination strategies.

Emerging molecular and cellular profiling tools have accelerated our understanding of perturbations induced by therapy within the PDAC TME.^6^ Single cell RNA-sequencing (scRNA-seq) allows measurement of the transcriptional profiles of individual cells directly from tumor specimens. Several groups have previously profiled the PDAC TME using scRNA-seq. These studies, however, largely characterize the TME during treatment in preclinical PDAC models, untreated human PDACs, or following exposure to standard-of-care chemotherapy.^7^^,^^8^^,^^9^ Neoadjuvant immunotherapy clinical trials provide large surgical biospecimens that enable examination of the TME in tissue directly from patients using these deep profiling technologies. This study reports a scRNA-seq dataset of tumor infiltrating leukocytes (TILs) obtained from patients with resectable PDAC treated with immunotherapy in three arms of our platform neoadjuvant-adjuvant trial (NCT02451982).

Previous multi-omics studies from this trial evaluated the benefit of our granulocyte-macrophage colony-stimulating factor (GM-CSF)-secreting allogeneic PDAC vaccine (GVAX). Notably, we found that GVAX induces T cell infiltration and formation of tertiary lymphoid aggregates (LAs) within PDAC tumors.^10^^,^^11^^,^^12^^,^^13^ However, LA formation in these patients was associated with the upregulation of immune checkpoint molecules, including programmed death-ligand 1 (PD-L1), on both myeloid and tumor cells.^12^ These findings provided rationale for a second treatment arm examining the addition of anti-PD1 (nivolumab) to GVAX, with the goal of relieving T cell exhaustion and enhancing productive anti-tumor T cell responses. In this second arm, we observed that increased abundance of intratumoral CD3^+^CD8^+^CD137^+^ T cells is associated with improved overall survival (OS > 2 years). This CD3^+^CD8^+^CD137^+^ population was found in greater numbers in the vaccine with anti-PD1 treatment arm, and comprised the largest population of cytotoxic CD8^+^GZMB^+^ T cells, linking CD137 upregulation to increased T cell function.^13^ Our data also suggest that anti-PD1 therapy indirectly activates CD8^+^ T cells by inducing alterations in the extracellular matrix (ECM) that may regulate CD8^+^ T cell trafficking.^13^

The platform trial design enables earlier arms to inform alternate combination therapies in additional arms. Based upon our previous study^13^ and supporting preclinical evidence for T cell activation with the addition of CD137 agonist in PDAC,^14^ we hypothesized that the addition of agonistic anti-CD137 would further enhance T cell activation and function when used in combination with GVAX and anti-PD1. CD137 is expressed at the cell surface by antigen-activated immune cells, particularly T cells,^15^ where it serves as a costimulatory molecule that enhances T cell proliferation, activation, and survival.^15^^,^^16^^,^^17^^,^^18^ Notably, CD137 agonism has a well-established history as an immunotherapeutic agent in cancer, as was recently reviewed in depth by Melero et al.^19^ Of relevance to our platform clinical trial, CD137 agonists are known to be synergistic with anti-PD1 in multiple preclinical mouse tumor models.^14^^,^^20^^,^^21^^,^^22^ Indeed, we observed that triple therapy with GVAX, anti-PD1, and CD137 agonist is not only safe but also associates with improved disease-free survival (DFS) and moderate pathologic responses in 3/10 patients just two weeks after a single neoadjuvant treatment.^23^ These types of responses are rare in human PDAC and provide promise for alternative treatment combinations to further enhance patient outcomes. Therefore, to examine the underlying immune cellular and molecular mechanisms of TME modulation in this three-arm trial, we leveraged our access to surgically resected PDAC tissue two weeks after neoadjuvant therapy to perform scRNA-seq with matched T cell receptor sequencing (TCR-seq) of purified TILs.

## Results

### scRNA-seq reveals distinct PDAC-infiltrating immune cell populations after treatment in the GVAX, nivolumab, and urelumab arm of a platform study

**Figure 1:**
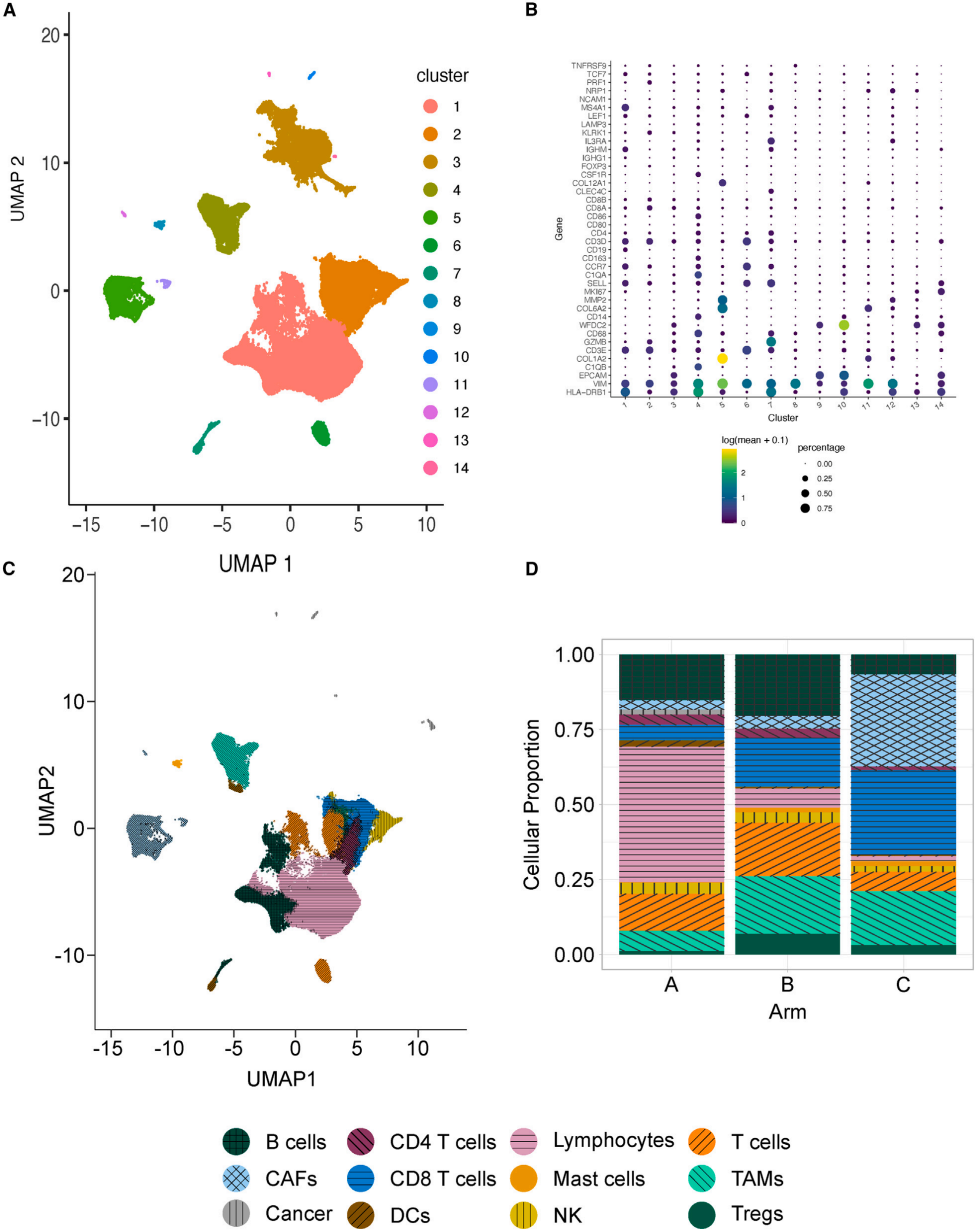
Summary of single-cell RNA-sequencing of PDAC-infiltrating leukocytes (A) UMAP depicting Leiden clustering of the dataset. (B) Dot plot showing genes and their expression levels used for cell annotations across clusters in the dataset. (C) UMAP summarizing the cell types annotated within the dataset based upon differential expression of marker genes between subclusters observed in each of the major clusters from C, displayed in Figure S1. (D) Barplots showing cellular proportions across treatment arms. Panels C and D were generated scatterHatch^24^ colors and patterns.

To examine CD137 agonist-induced alterations to TIL in patients treated in our combination immunotherapy clinical trial,^23^ we purified TILs from resected tumors (see STAR Methods) from seven patients receiving cyclophosphamide (Cy)/GVAX alone (Arm A), four patients receiving the combination of Cy/GVAX and anti-PD1 (nivolumab, Arm B), and four patients receiving Cy/GVAX, anti-PD1, and CD137 agonist (urelumab, a human IgG4 agonistic monoclonal antibody, Arm C) (Table 1). In this trial each patient with clinically resectable PDAC received their neoadjuvant treatment two weeks prior to surgery. TILs were analyzed by scRNA-seq with matched scTCR-seq (10× Genomics). Overall, we obtained high-quality transcriptomes from 112,247 cells. Further analysis with Leiden clustering revealed 14 distinct clusters (Figure 1A), and subsequent annotations showed populations of B cells, CD4^+^ T cells, Tregs, CD8^+^ T cells, mixed lymphocytes (consisting of naive, B cell, and T cell marker genes), mixed T cells (consisting of naive, CD4^+^, and CD8^+^ T cell genes), natural killer cells (NK), tumor associated macrophages (TAMs), dendritic cells (DCs), mast cells, cancer associated fibroblasts (CAFs), and cancer cells (Figures 1B and 1C). Details of genes used to annotate each cluster and number of cells within each population can be found in Figure S1, S2, and Table S1. Additionally, a single cluster was annotated as dying cells based on consistently high percentages of mitochondrial gene expression across cells relative to other clusters and was removed from analysis (Figure S3). Cellular proportions varied across treatment arms, with most mixed lymphocytes coming from a single patient in Arm A, B cells and Tregs from Arm B, mast cells from Arms B and C, and CAFs and CD8^+^ T cells from Arm C (Figure 1D). Addition of anti-PD1 to GVAX (Arm B) demonstrated an apparent increase in the abundance of both PDAC-infiltrating Tregs and CD8^+^ T cells, while inclusion of CD137 agonism showed reduced Treg and increased CD8^+^ T cell abundance. These observations, however, may not represent the overall intratumoral abundances of infiltrating TILs, as we only sample a small portion of each patient’s tumor in these analyses.

**Table 1:** Clinical features and cell counts of Patients included in single-cell analyses

Patient	Arm	Age at Surgery	Sex	OS	Number of cells in dataset
P-45	Arm A	<65	F	>2 years	45102
P-44	Arm A	≥65	F	<2 years	12221
P-2	Arm A	<65	M	>2 years	3806
P-42	Arm A	<65	F	>2 years	10361
P-6	Arm A	<65	M	<2 years	5162
P-20	Arm A	≥65	F	>2 years	1673
P-33	Arm A	≥65	F	>2 years	4577
P-24	Arm B	≥65	M	>2 years	1393
P-32	Arm B	<65	M	>2 years	1297
P-17	Arm B	<65	M	>2 years	2522
P-3	Arm B	<65	M	>2 years	1224
P-25	Arm C	≥65	F	>2 years	3567
P-23	Arm C	<65	F	<2 years	3455
P-46	Arm C	≥65	M	>2 years	4867
P-40	Arm C	<65	F	>2 years	11020

Age at Surgery is reported as age less than 65 years or greater than or equal to 65 years. OS, overall survival; M, male sex; F, female sex.

### Differential expression analysis reveals transcriptomic alterations in CD8^+^ T cell cytoskeletal and ECM-interacting components that are largely driven by anti-PD1

**Figure 2:**
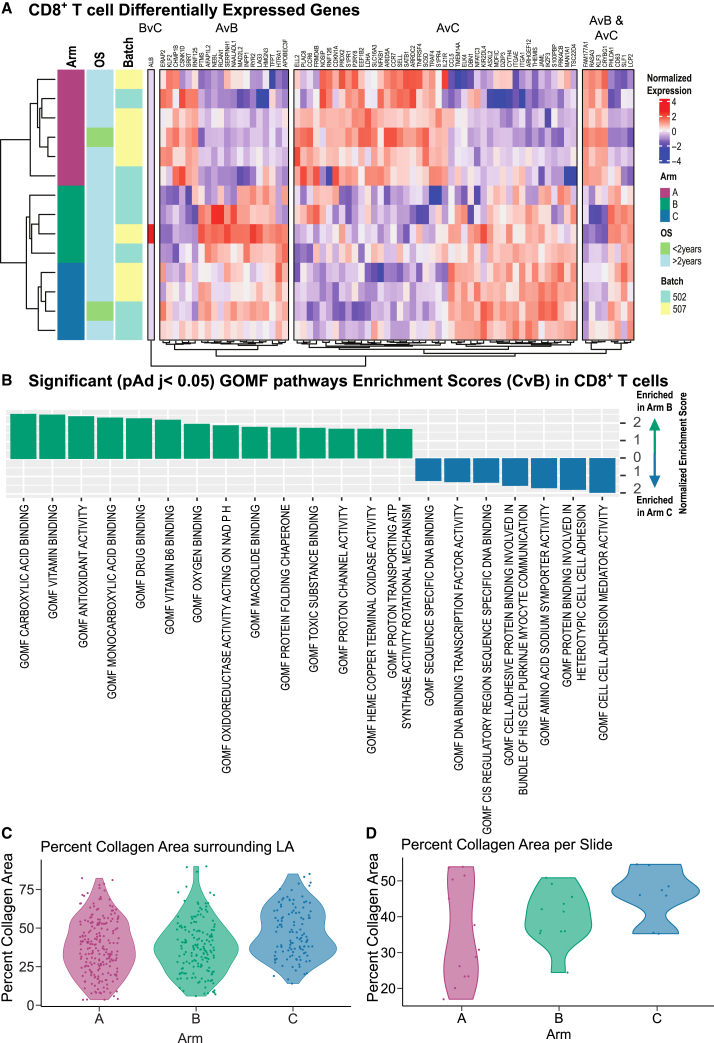
Differential expression of CD8^+^ T cells across treatment arms (A) Heatmap of the normalized Variance Stabilizing Transformation (VST) expression of differentially expressed genes from the following comparisons indicated on the right of heatmap: Arm A vs. B, Arm B vs. C, Arm A vs. C, and genes present in both the Arm AvB and AvC comparisons. Genes are grouped into the bins from which comparison they arose. (B) Gene set enrichment barplot from GOMF of Arms B vs. C. Positive normalized enrichment indicates higher presence in Arm B samples, while negative normalized enrichment scores indicate higher presence in Arm C samples. (C) Violin plot of percent CA surrounding each LA by treatment arm. Overlayed points represent each measured LA. (D) Violin plot of percent CA of the entire tissue section for each patient. Overlayed points represent each measured slide.

CD8^+^ T cells are the intended target for therapeutic antibody modulation when they express PD1 and CD137. We therefore hypothesized that we could detect treatment-induced changes in gene expression within our CD8^+^ T cell population. To assess this, we performed pseudobulk differential gene expression analysis of tumor-infiltrating CD8^+^ T cells across all three treatment arms. We found significant gene expression differences within the CD8^+^ T cell subset, particularly between GVAX monotherapy (Arm A) and both the dual GVAX+anti-PD-1 (Arm B) and triple GVAX+anti-PD-1+CD137 agonist (Arm C) therapies (Figure 2A; Table S2). Furthermore, the differentially expressed genes from both Arms B and C as compared to Arm A were similar, as visualized in the heatmap in Figure 2A. Indeed, several genes are significantly downregulated in Arms B and C as compared to Arm A (*FAM177A1*, *RASA3*, *KLF3*, and *CRYBG1*) and, conversely, several genes are upregulated in both Arms B and C as compared to Arm A (*PHLDA1*, *CD63*, *SLF1*, *LCP2*).

Examination of the differentially expressed genes overlapping between Arms B and C as compared to Arm A revealed cytoskeletal and ECM-interacting components, consistent with our previous findings from bulk RNA-seq analysis,^13^ as well as regulators of T cell function. For example, the genes downregulated in both Arms B and C include *CRYBG1*, also referred to as *AIM1*, an actin binding protein reported to suppress remodeling of the cytoskeleton^25^; *KLF3*, a transcription factor associated with a quiescent T cell state^26^; *RASA3*, an inhibitor of LFA-1 that enables T cell migration into tissues^27^; and *FAM177A1*, which was recently reported to function as a negative regulator of IL1B signaling.^28^ Genes upregulated in both Arms B and C include *LCP2*, which is involved in TCR signal transduction^29^; *CD63*, which is classified as a costimulatory molecule that promotes both activation and proliferation of T cells^30^; and *PHLDA1*, which may have inhibitory functions as its expression within exhausted tumor infiltrating CD8^+^ T cells in both HCC and NSCLC is associated with poor prognosis.^31^^,^^32^ Notably, another exhaustion marker, *LAG3*, is upregulated in Arm B when comparing Arms A and B, and upregulation of both *PHLDA1* and *LAG3* demonstrate that alternate exhaustion molecules are expressed by CD8^+^ T cells after anti-PD1 therapy. When comparing Arm B to Arm C, however, only *ALB* is differentially expressed, and this is driven by high expression in cells from a single patient in Arm B. Taken together, these findings suggest that gene expression changes within CD8^+^ T cells across treatment arms are induced by anti-PD1, with negligible alterations upon addition of CD137 agonist.

We next performed pathway analysis (Gene Ontology Molecular Function, GOMF) for these CD8^+^ T cells to further assess the molecular mechanisms associated with gene expression changes across treatment arms. When compared to Arm A, Arm B is associated with elevated enrichment of protein activity as well as changes in electron transfer. Additionally, comparing Arms B and C reveals changes in sequence specific DNA binding including by transcription factors and at *cis*-regulatory regions in Arm C, as well as enrichment in cell adhesion molecules and mediator activity (Figure 2B). These data suggest that while anti-PD1 is associated with gene expression changes detected in CD8^+^ T cells across treatment arms, anti-CD137 agonism further modifies the function of these cells by increasing cell adhesion molecule expression and thus interactions with other cells and ECM components within the TME.

To explore whether ECM alterations are detectable at the protein level across treatment arms, we employed Masson’s trichrome staining for collagen, the most abundant protein in the ECM, on tissue sections from post-treatment tumors from 13 patients in Arm A, 12 patients in Arm B, and 9 patients in Arm C (Table S3). Lymphoid aggregates (LAs), defined as regions containing abundant immune cell populations including T cells, were annotated for each section using HALO software (see STAR Methods). Percent collagen area (CA) was next calculated as the percent of collagen positive pixels averaged across regions of interest extending to a radius of 200μm from the annotated LA (see STAR Methods and Figure S4A). Using this approach, we found that the area surrounding LAs from Arm C tend to have a higher percent CA than those from Arms A and B (Figure 2C), suggesting that patients treated with the combination of GVAX, anti-PD1, and CD137 agonist have a denser intratumoral collagen network surrounding their LAs than those treated with GVAX or GVAX and anti-PD1. This trend holds true when examining percent CA across the regions of interest individually (Figure S4B) and by patient (Figure S4C). Indeed, when exploring percent CA across the entire tissue section, Arm C generally has a higher percent CA than either Arms A or B (Figure 2D). While the sample sizes are small and these trends do not reach statistical significance, these data suggest that the ECM, specifically collagen density, varies across treatment arms, supporting our finding that CD8^+^ T cells differentially express ECM-interacting genes by arm.

### Integrated single-cell TCR and RNA-seq analysis reveals a population of activated and clonal tumor-infiltrating CD8^+^ T cells in patients receiving the combination of GVAX, anti-PD1, and CD137 agonist

**Figure 3:**
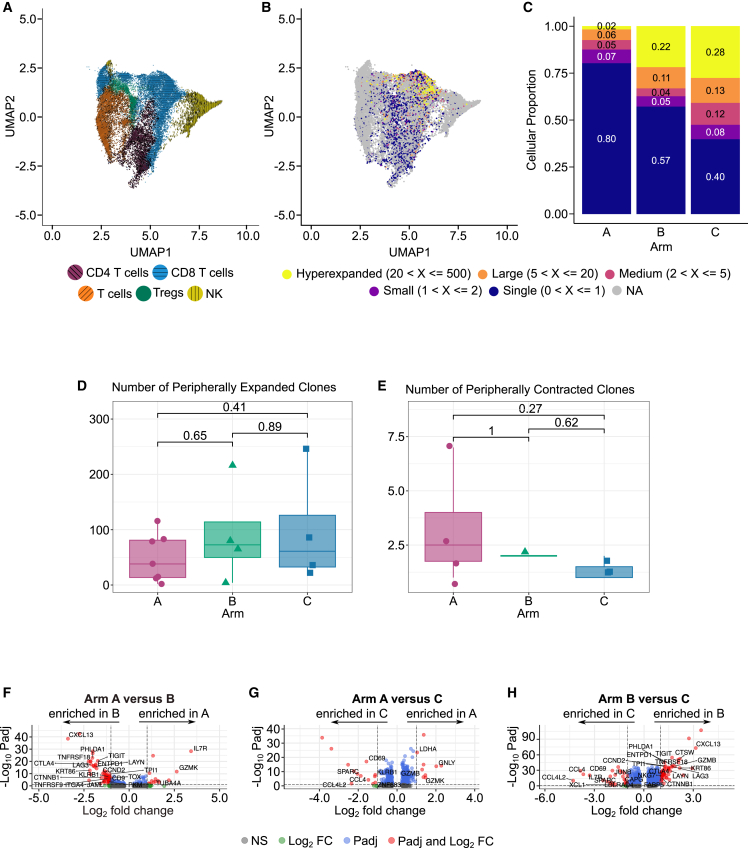
Addition of anti-PD1 and CD137 agonist to Cy/GVAX is associated with hyperabundant and clonal tumor-infiltrating CD8^+^ effector T cells with improved function (A) UMAP of the major T cell cluster from scRNA-seq (subset from Figure 2A). (B) UMAP of the cells from panel A colored by T cell expansion status calculated from matched scTCR-seq. (C) Stacked barplots showing the proportions of T cells from each Arm belonging to hyperexpanded, large, medium, small, or singlet clones. Overlayed numbers are the proportion of T cells belonging to the clone types. (D and E) Boxplots showing the number of expanded T cell clones (D) and contracted T cell clones (E) in the peripheral blood after therapy by Arm. Boxplots display the 25^th^ percentile (top), median (middle), and 75^th^ percentile (bottom) T cell clone numbers within each Arm with whiskers extending to 1.5 times the interquartile range and overlayed with individual sample measurements as points. Wilcoxon test *p*-values are displayed above. (F–H) Volcano plots of differential expression analyses using a MAST test of expanded CD8^+^ T cells comparing Arms A and B (F), Arms A and C (G), and Arms B and C (H). LFC: log fold change, Padj: Bonferroni adjusted *p* value.

Although we did not detect major gene expression changes between PDAC-infiltrating CD8^+^ T cells from patients receiving GVAX and anti-PD1 as compared to those also receiving the CD137 agonist, we hypothesized that the triple therapy may enhance clonal abundance of intratumoral CD8^+^ T cells. Therefore, using our matched scTCR-seq data, we quantified the abundance of T cell clones within the major T cell cluster (Figures 3A–3C). We found a population of clonal T cells, consisting of at least 20 T cells with the same TCR, almost entirely within the CD8^+^ T cell population (86.7% from Arm A, 81.9% from Arm B, and 98.0% from Arm C, Figure 3B; Figure S3A). Both the dual (Arm B) and triple therapy (Arm C) are associated with an increase in these hyperabundant clonal T cells as compared to GVAX alone (Figure 3C). These findings indicate that anti-PD1 therapy increases the abundance of clonal PDAC-infiltrating T cells, an observation that is enhanced by the addition of CD137 agonist. Furthermore, T cell clones with at least 5 cells containing the same TCR largely demonstrate predicted cell cycle phases across the entire cell cycle, suggesting that these cells may have clonally expanded in response to therapy (Figures S5B and S5C). However, given that our TILs are sampled from a single timepoint, we cannot conclude whether the observed hyperabundant clones expanded in response to therapy or rather represent a pre-existing population of intratumoral clonally expanded T cells.

Unfortunately, pre-treatment biopsies had too few TIL to assess changes after therapy. Therefore, to explore the dynamics of these hyperabundant PDAC-infiltrating CD8^+^ T cell clones before and after therapy, we next performed bulk TCR-seq on peripheral blood mononuclear cells (PBMCs) from all patients in our single-cell cohort at baseline (pre-treatment) and at time of surgical resection two weeks after treatment (post-treatment, matched timepoint to the scRNA-seq TILs) using Adaptive Biotechnologies immunoSEQ TCRB assay. Overall, we found no significant differences between arms or timepoints in overall number of unique T cell clones, T cells, or clonality (Figures S6A–S6C). The majority of hyperabundant (>20 cells) and large (>5 cells) PDAC-infiltrating CD8^+^ T cell clones were detected in the periphery (9/9 hyperabundant and 57/60 large, Table S4). To quantify how many clones expand peripherally after treatment initiation, we employed a Fisher’s Exact Test to compare the abundance of all T cell clones pre and post treatment.^33^ We found that the number of peripherally expanded clones after therapy did not vary across treatment arms (Figure 3D), nor were any of the hyperabundant or large intratumoral clones identified as expanded in this analysis (Table S4). It remains a possibility that rather than expanding peripherally in response to treatment, relevant T cells may instead traffic to the tumor after therapy and thereby decrease in abundance in the periphery. To explore this possibility, we also applied the Fisher’s Exact Test to quantify how many clones significantly contract after therapy. We found very few clones that contract by this analysis (<10 per patient), and again there were no detectable differences across treatment arms (Figure 3E; Table S4). To further assess the dynamics of the hyperabundant and large PDAC-infiltrating T cell clones from the single-cell data, we repeated these Fisher’s Exact Tests just on this cellular subset. Once again, we found that most of these T cells were not identified as expanded in the periphery after treatment by this analysis (2/9 hyperabundant and 8/60 large clones considered expanded, Table S4), nor were any contracted. Taken together, these data suggest that peripheral T cell abundances and dynamics do not reflect the intratumoral TCR repertoire in our cohort.

We next hypothesized that the clonally abundant CD8^+^ T cells from our scRNA-seq TIL dataset are more likely to be involved in anti-tumor immune responses and would thus express more activated gene expression profiles. We therefore further evaluated the subset of hyperabundant and large CD8^+^ T cell clones (more than five cells per clone). This CD8^+^ T cell population consists of cells contributed by 3 patients from Arm A and all 4 patients each from Arms B and C (11 patients total, Table S5). We next performed single-cell differential expression analysis using the MAST^34^ test on this subset of expanded CD8^+^ T cells across all combinations of treatment arms. We used this approach rather than pseudobulking and DESeq2 as we did for the differential expression analyses comparing treatments of other cell populations because of the low abundance of expanded CD8^+^ T cells within individual patients, and therefore note that patient-specific effects may impact this analysis. Despite these limitations, we found significant differentially expressed genes across all comparisons (Figures 3F–3H; Table S6).

Genes upregulated in Arm A as compared to Arm B include *IL7R*, a key receptor for the survival and proliferation of naive and memory T cells,^35^ and *GZMK*, a cytotoxic molecule associated with poor prognosis in colorectal cancer.^36^ Genes enriched in Arm B as compared to Arm A include *CXCL13*, an important chemokine in tertiary lymphoid structure development^37^ that was shown to be a marker of response to anti-PD1 therapy when expressed by tumor infiltrating CD8^+^ T cells in NSCLC,^32^ as well as the activation markers *TNFRSF9* (CD137/4-1BB) and *KLRB1* (CD161). Notably, this CD137 upregulation with anti-PD1 therapy fits with our previous findings,^13^ while expression of *KLRB1* is associated with reactivated cytotoxic CD8^+^ T cells^38^ within the TME and correlates with better outcomes in several different cancers.^39^ Additional genes upregulated in Arm B as compared to Arm A include *PHLDA1*; the exhaustion markers *CTLA4*, *TIGIT*, *LAG3*, *TOX*,^40^ *LAYN*^30^*,* and *ENTPD1* (CD39)^41^; and the costimulatory receptor *TNFRSF18* (GITR), which are all associated with T cell activation upon antigen stimulation. Numerous cytoskeletal and ECM-interacting molecules were also differentially expressed (*KRT86*, *CCND2*, *CTNNB1*, *JAML*, *TUBA4A*, *CD9*, and *ITGA4*), supporting our previous findings after anti-PD1 therapy,^13^ as well as metabolism-associated *TPI1* and *PKM*. These changes suggest a transition of CD8^+^ T cells from a GVAX-responsive low activity status to a more activated status when anti-PD1 is added, albeit with further induction of T cell exhaustion.

Comparing hyperabundant CD8^+^ T cells from the TILs of patients in Arms A to those in Arm C revealed downregulation of genes in Arm C including the cytotoxic molecules *GZMB*, *GNLY*, and *GZMK*, and the metabolic enzyme *LDHA,* suggesting potential alterations in effector function induced by CD137 agonism. Arm C showed enhanced expression when compared to Arm A of the activation markers *CD69* and *KLRB1* (CD161), the cytokines *CCL4* and *CCL4L2* which recruit immune cells to inflammatory sites, *ZNF683* (Hobit), and the ECM glycoprotein *SPARC* (osteonectin). These results indicate that the addition of anti-PD1 and CD137 agonist to GVAX enhances CD8^*+*^ T cell activation. When comparing Arms B to C, we found genes enriched in Arm B including the chemokine *CXCL13*; the anti-inflammatory molecule *PHLDA1*; the cytolytic molecules *CTSW*, *GZMB*, and *NKG7*, the latter of which has been recently reported as an immunotherapeutic target to improve CD8^+^ T cell cytotoxicity^42^; the exhaustion markers *CTLA4*, *TIGIT*, *LAG3*, *LAYN,* and *ENTPD1* (CD39); metabolic genes *TPI1* and *FABP5*; the cytoskeletal and ECM interacting molecules *CCND2*, *KRT86*, *CTNNB1*; and *TNFRSF18* (GITR). Arm C genes, in contrast, were enriched for the activation associated markers *CD69*, *JUNB*, and *XCL1*^43^; cytoskeletal and proliferation markers *DDIT1* (REDD1), *CAPG*, and *LDLRAD4*; cytokines *CCL4* and *CCL4L2*; the ECM glycoprotein *SPARC* (osteonectin); and *IL7R*. Overall these data suggest that addition of a CD137 agonist to GVAX and anti-PD1 therapy induces cytoskeletal and ECM alterations, reduces exhaustion markers, and enhances tumor-infiltrating CD8^+^ T cell activation and proliferation.

### Immune checkpoint inhibition with anti-PD1 is associated with reduced CD8^+^ T cell signaling through CCR7 and IL7R while addition of a CD137 agonist enhances immunosuppressive TAM signaling through TREM2

**Figure 4:**
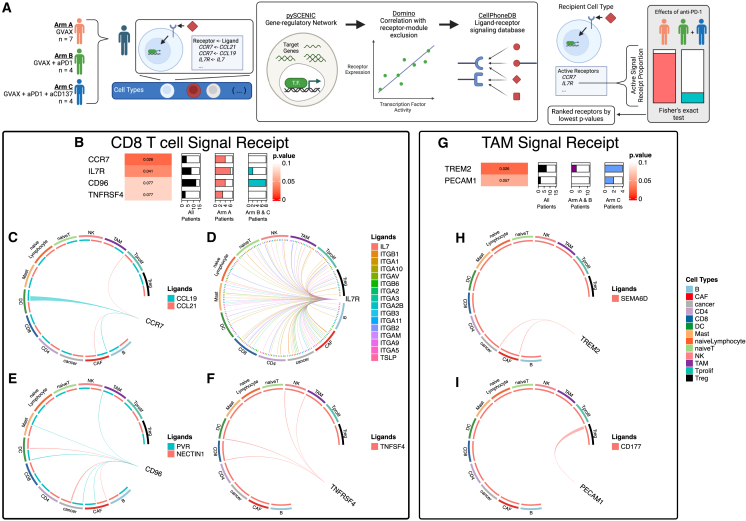
Combination immunotherapy approaches elicit alterations in the interaction of CD8^+^ T cells and TAMs with other cells in the TME (A) Workflow of cellular communication inference using Domino and comparing the proportions of patients with active signal receipt between treatment groups. Briefly, the Domino method infers active signaling based on identifying correlation of receptor expression with downstream transcription factor regulons and coordinated gene expression of theses receptors with cognate ligands expressed by other cells, indicating a candidate cell-cell signaling event. Inferred interactions are tallied across patients in each trial arm, and a Fisher’s exact test is employed to rank the extent to which ligand-receptor cellular interactions differ between treatment arms. (B) Ranking of receptors in CD8^+^ T cells prioritized from our differential ligand-receptor analysis between patients from treatment arm A relative to patients in both treatment arms B and C. The values shaded blocks represent *p*-values from a Fisher’s Exact Test comparing the frequency of patients with active signal receipt as determined using Domino, with darker color denoting lower *p*-values. The colored bars show the frequency of active signal receipt among all patients (black), patients in arm A (light red), or arms B and C (teal) tallied as described in A. (C–F) Circos plots are used to show the ligands in the cell types found to signal with the receptors in CD8^+^ T cells in trial arm A from the differential ligand-receptor analysis. The width of chords corresponds to the mean normalized expression of the ligand gene within a cell type in the data for all patients from trial arm A, representative of the putative strength of signaling from that ligand in the given cell type to the receptor in CD8^+^ T cells. Because each receptor may have multiple ligands, chords and the inner annotation are colored based on all candidate ligand genes that can signal to a receptor. The circle is divided into cell types, indicated by the labels for each outer arc. Circos plots are divided by each candidate receptor prioritized from the differential analysis in B, with ligands associated with (C) CCR7, (D) IL7R, (E) CD96, and (F) TNFRSF4. (G) Ranked signal receipt in TAMs between the patients in treatment arms A and B relative to patients from treatment arm C. The colored bars show the frequency of signaling among all patients (black), patients from arms A and B (purple), or patients from arm C (light blue). (H) Circos plot from the single-cell data for patients in trial arm C of ligands associated with TREM2 and with (I) PECAM1.

Given the marked gene expression changes induced within CD8^+^ T cells with anti-PD1 therapy and CD137 agonism, we next examined accompanying alterations in cellular communication via ligand-receptor interactions between cell subsets within the PDAC TME. We used the cellular communication inference tool Domino^44^ to infer activation states of receptors from our scRNA-seq data and assess the expression of ligands capable of instigating their activation among other cell types. To identify cellular communication interactions that differentially occur due to treatment, we expanded this computational method to compare the inter-cellular networks functioning in the different treatment arms (Figure 4A). Briefly, Domino identifies active signaling via ligand-receptor pairs between cell types in an scRNA-seq dataset. Here, we present an innovative application of Domino for the assessment of differential signals among patients by applying Domino to scRNA-seq data for each patient and performing a Fisher’s exact test upon the inferred signals to compare which ligand-receptor pairs differ between patients from the distinct treatment groups. To specifically identify cellular interactions related to immune checkpoint inhibition with anti-PD1, signaling through expressed receptors was compared between patients in Arm A (*n* = 7) and a combined grouping of patients from Arms B and C (*n* = 8). Among CD8^+^ T cells from these patients, the receptors more commonly active in patients from Arm A relative to the patients from the other trial arms were CCR7 (A: 4/7; B & C: 0/8) and IL7R (A: 6/7; B & C: 2/8) (Figure 4B). CCR7 is highly expressed on naive and memory T cells and has important functions as a chemokine receptor in the guidance of lymphocytes and dendritic cells to lymph nodes and tertiary lymphoid structures.^45^^,^^46^ The chemokines that instigate this immune cell homing, *CCL19* and *CCL21*, may be expressed by endothelial cells in high endothelial venules or mature dendritic cells in the case of *CCL19*.^45^ Indeed, we found *CCL19* to be differentially expressed by intratumoral dendritic cells as compared to all other cell types (Figure 4C). Additionally, IL7R signaling, important for the survival and proliferation of naive and memory T cells as previously discussed, was also reduced in Arms B and C as compared to Arm A. Within our data, *IL7*, a ligand for *IL7R*, was detected in Tregs and cancer cells (Figure 4D). The high frequency of patients with CD8^+^ T cells exhibiting signaling via these receptors in Arm A compared to the other treatment arms suggests a preponderance of T cells dependent on IL-7 signaling for survival and exhibiting homing to lymphoid structures and awaiting activation into effector states. The inclusion of anti-PD1 may decrease the relative frequency of these T cells, as clonally expanded T cells comprise a greater proportion of PDAC-infiltrating T cells in both Arms B and C (Figure 3C).

Signaling via CD96 was more frequent among CD8^+^ T cells from patients in Arms B and C (8/8) as compared to Arm A (4/7) (Figure 4B). CD96 belongs to the immunoglobulin superfamily and acts as a co-inhibitory receptor that competes with the co-stimulatory receptor CD226 for the shared ligands CD155 (*PVR*) and CD211 (*NECTIN1*), both of which are expressed on tumor and myeloid cells.^47^ CD96 has been the subject of investigation as an additional target of immune checkpoint inhibition in cancer immunotherapy.^48^ The relative increase in active signal receipt via CD96 may represent compensatory means of tumor immune evasion as cancer cells, CAFs, TAMs, DCs, and mast cells were all found to express *PVR* and *NECTIN1* within our dataset, both of which are capable of inducing immunosuppression in CD8^*+*^ T cells (Figure 4E). *TNFRSF4*, encoding OX40, is a member of the tumor necrosis factor receptor superfamily. Signaling through OX40 promotes T cell activation, prevention of apoptosis, and promotion of T follicular helper differentiation in a B follicle context.^49^^,^^50^ Signaling via *TNFRSF4* was found at greater frequency among CD8^+^ T cells from patients in Arm A (4/7) as compared to Arms B and C (0/8) (Figure 4B). The prevalence of this co-stimulatory signaling in CD8^+^ T cells from patients in Arm A along with the signatures of potentially more naive T cell states including increased signaling via CCR7 may suggest decreased frequency of cellular interactions intended to prime and activate T cells that have reached the TME upon receipt of anti-PD1 therapy. Both DCs and TAMs from the tumor exhibit some expression of *TNFSF4* (Figure 4F), encoding OX40 Ligand (OX40L), suggesting the increased frequency of TNFRSF4 activation could be due to interactions with these cell types.

TAMs also exhibit notable differences in the receipt of ligand-receptor signals, particularly after the inclusion of CD137 agonism. Arm C was associated with increased signaling via TREM2 (A & B: 3/11; C: 4/4) and CD31 (*PECAM1*) (A & B: 0/11; C: 2/4) (Figure 4G). *TREM2*+ macrophages have been found to accumulate in human tumors,^51^ with infiltration of TREM2/APOE/C1Q expressing macrophages serving as a potential biomarker for disease recurrence in clear cell renal carcinoma.^52^ Macrophages characterized by the expression of TREM2/APOE/C1Q are also one of predominant subtypes in both human and murine PDACs.^53^ Furthermore, TREM2 expression by suppressive monocytes has been shown to inhibit CD8^+^ T cell function by enhancing exhaustion and PD-1 blockade resistance.^54^ Coordinated ligand-receptor expression changes through TREM2 among TAMs from patients in Arm C suggest a role for CD137 agonism in promoting activation of a macrophage checkpoint. Within our data, we found expression of *SEMA6D* (semaphorin 6D) by cancer cells, a ligand of TREM2 involved in axon guidance,^55^ suggesting that cancer cells may serve as a source of instigating ligands in this signaling pathway (Figure 4H). *PECAM1* encodes platelet endothelial cell adhesion molecule-1 (PECAM-1), an adhesion and signaling molecule expressed by endothelial cells as well as leukocytes and other blood cells that could be indicative of vasculogenesis within the TME. Furthermore, CD31 induces inhibitory signals when engaged in immune cells,^56^ and active signaling in macrophages can trigger polarization changes from pro-inflammatory to reparative TAMs.^57^ Such a transition in macrophage polarization could represent a further means of immune evasion by PDAC. This is further supported through the expression of *CD177*, the activating ligand of CD31, by immunosuppressive Tregs (Figure 4I).

### Anti-PD1 induces transcriptomic alterations in TAM metabolism, function, and ECM-interactions that are enhanced by CD137 agonism

**Figure 5:**
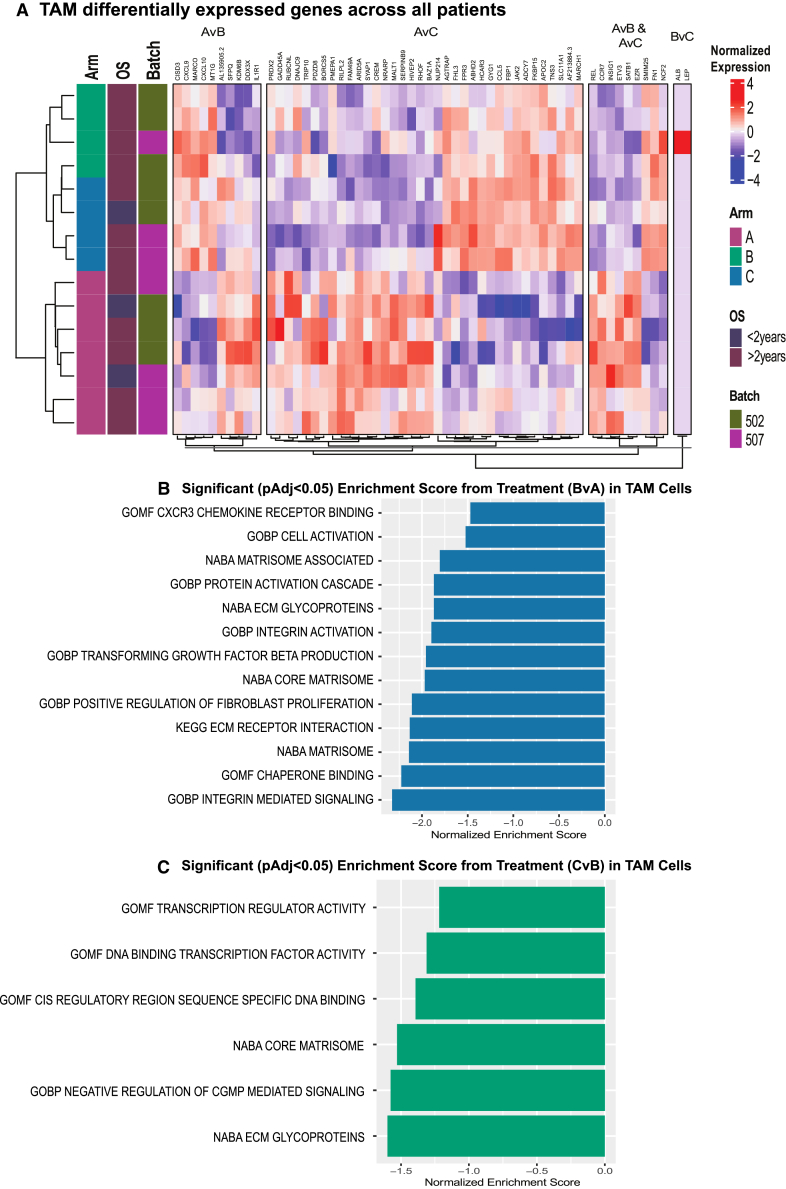
Differential expression of TAMs across treatment arms (A) Heatmap of normalized VST expression of differentially expressed genes from the following comparisons indicated on the right of heatmap: Arm A vs. B, Arm B vs. C, Arm A vs. C, and genes present in both the Arm AvB and AvC comparisons. Genes are grouped into the bins from which comparison they arose. (B) Gene set enrichment barplot comparing Arms A and B. Positive normalized enrichment indicates higher presence in Arm A samples, negative normalized enrichment scores indicate higher presence in Arm B samples. (C) Gene set enrichment barplot comparing Arms B and C. Positive normalized enrichment indicates higher presence in Arm B samples, negative normalized enrichment scores indicate higher presence in Arm C samples.

Since CD137 agonism measurably alters signaling through both TREM2 and PECAM-1 in TAMs, we examined TAM gene expression changes between each treatment arm using our scRNA-seq TIL data. As we found for CD8^+^ T cells, most differentially expressed genes in TAMs are detected after the addition of anti-PD1, again with several of the same genes overlapping between both Arms B and C as compared to Arm A (Figure 5A; Table S7). In addition to these overlapping genes, we also detected differentially expressed genes exclusive to Arms B and C as compared to Arm A. Although specific to either Arm B or C when comparing to Arm A, the expression profiles of these genes remain markedly similar between Arms B and C, further highlighting the impact of anti-PD1 on gene expression alterations within TAMs. The differentially expressed genes upregulated in Arm A as compared to Arms B and C include *REL*, a subunit of NFkB for which inhibition has been shown to block cancer growth in mice^58^ and is thus posited to be a potential myeloid checkpoint molecule; the previously discussed *CCR7*; and *EZR*, which encodes a protein that is crucial for cytoskeletal and ECM alterations and interactions that is associated with tumor-promoting TAMs.^59^ *FN1*, encoding another ECM protein that was associated with tumor-promoting macrophages in gastric cancer,^60^ was upregulated in Arms B and C, as was the metabolic regulator *INSIG1*. Altogether these findings suggest that anti-PD1 induces potent gene expression changes that alter TAM function, metabolism, and ECM interactions via modulation of cytoskeletal and ECM components.

When comparing Arms A and B, we found upregulation of *IL1R* in Arm A, which has been shown to both maintain immunosuppressive TAM phenotypes and promote tumor growth via upregulation of PD1 in melanoma,^61^ while *CXCL9* and *CXCL10*, both ligands for CXCR3, were upregulated in Arm B, for which expression in TAMs is associated with effector CD8^+^ T cell infiltration into various solid tumors and are markers of response to ICIs.^62^^,^^63^^,^^64^^,^^65^ Also upregulated in Arm B is *MARCO*, a gene encoding a scavenger receptor that has been associated with immunosuppressive TAMs in non-small cell lung cancer^66^ and, notably, with poor prognosis in patients with PDAC.^67^ Therapeutic targeting of MARCO has also been proposed to enhance anti-tumor immune activity in several solid tumors.^68^ When comparing Arms A and C, we find upregulation of genes in Arm A corresponding to immunosuppressive TAMs, including *ARID5A*, for which repression is pro-tumorigenic in lung cancer,^69^ and *CREM*, which is associated with tumor-promoting TAMs in gastric adenocarcinoma,^70^ while genes upregulated in Arm C include those associated with metabolism (*FBP1*, *APOC2*, *AF213884.3*); *CCL5*, which has been shown to have CD8^+^ T cell agonism activity in PDACs^71^; *SLC11A1*, the expression of which is associated with immunosuppressive TAMs in colorectal cancer^72^; and cytoskeletal and ECM-associated molecules (*FKBP15*, *TNS3*). In contrast, when comparing Arms B and C, we only detect two differentially expressed genes, *ALB* and *LEP*, both of which are highly expressed in cells from a single patient from Arm B. Overall, these analyses suggest that addition of an anti-CD137 agonist to anti-PD1 does not broadly alter the gene expression profile of TAMs. In contrast to the differential signaling analysis, *TREM2* is not statistically significantly differentially expressed in TAMS between the trial arms. However, CD137 agonism is associated with increased TREM2 signaling from CAFs and neoplastic cells as described above, and may further alter TAM function, ECM, and metabolism through cell-cell communication resulting from that cell type rather than changes in the *TREM2* expression within all macrophages.

Gene set enrichment analysis comparing Arm A to Arm B showed increased interactions with the ECM (KEGG ECM-receptor interaction), CXCR3 chemokine receptor binding, chaperone binding, cell activation (GOBP cell activation), integrin mediated signaling pathways (GOBP), positive regulation of fibroblast proliferation (GOBP), TFG-B production (GOBP), integrin activation (GOBP), matrisome and ECM glycoprotein interactions (NABA), and protein activation cascades (GOBP) after addition of anti-PD1 (Figure 5B). These data further support a role for anti-PD1 in modifying TAM cytoskeletal components and ECM interaction, and TAM function via diverse signaling and activation pathways. To further assess the impact of CD137 agonism on TAM phenotype, we used pathway analysis to compare Arms B and C and observed increases in transcription regulator activity (GOMF), DNA binding (GOMF), negative regulation of CGMP mediated signaling (GOBP), core matrisome (NABA) and ECM glycoproteins (NABA) in Arm C (Figure 5C). Thus, CD137 agonism may contribute to shifts in TAM function and ECM interactions beyond the effects induced by anti-PD1.

## Discussion

The single-cell analyses from a human neoadjuvant clinical trial conducted in this study suggest distinct immune cell changes within the PDAC TME after the addition of anti-PD1 to a T cell inducing vaccine, GVAX, with or without CD137 agonist. As previously reported, we found that anti-PD1 modifies CD8^+^ T cell cytoskeletal and ECM-interacting components.^13^ Notably, anti-PD1 is also associated with an increased population of clonal and abundant CD8^+^ T cells with further observed increases after the addition of CD137 agonist. Stratification of CD8^+^ T cells from hyperabundant subclones enabled discernment of gene expression-level changes that were attributable to anti-PD1 given with GVAX versus with the addition of CD137 agonist. These included shifts in cytoskeletal and ECM-interacting molecules associated with both anti-PD1 and CD137 agonism, and CD137 agonism-associated enhancement of activation and abundance of clonally expanded CD8^+^ T cells. Thus, addition of CD137 agonist to GVAX and anti-PD1 may activate a subset of CD8^+^ T cells with concomitant changes in cytoskeletal components and ECM interactions. We also found that CD137 agonism may promote immunosuppressive signaling through TREM2 in TAMs. This altered signaling occurs along with gene expression changes indicative of shifts in TAM metabolism, ECM interactions, and function. These contradictory CD137 agonist functions suggest a compensatory mechanism by which the PDAC TME responds to a markedly activated T cell response. Incorporating additional TAM modulators may therefore improve efficacy of immunotherapy in PDACs, and supports recent preclinical studies of combination immunotherapies including myeloid-modulating inhibitors that enhance anti-tumor immunity and survival in murine models of PDAC.^65^^,^^73^^,^^74^

This study employed an innovative statistical approach to assess differential cellular communication interactions that occurred across multiple treatments. Current approaches to differential comparisons of ligand receptor networks, including NicheNet,^75^ base comparisons on the differential expression of ligands between user-defined niches of potentially interacting cells. The methods presented here leverage Domino’s^44^ capacity to infer receptor activation within annotated cell type clusters based on the expression of downstream targets of activated transcription factors across multiple patients in each treatment arm. This analysis revealed alterations in CD8^+^ T cell and TAM signaling phenotypes across treatments that were supported by complementary TCR repertoire analysis demonstrating an anti-PD1 treatment-associated population of abundant and clonal T cells exhibiting effector phenotypes, as well as differential expression of genes involved in macrophage function, cytoskeletal, and ECM interacting components dependent on CD137 agonist treatment.

Assessing molecular changes within individual cells of the TME after PDAC immunotherapy is essential for determining mechanisms of treatment resistance. Notably, this study generated a scRNA-seq atlas of three additive immunotherapy regimens in resectable PDAC. These data therefore represent a valuable resource to further dissect the contribution of immunotherapy to immune cell modulation directly within PDAC tumors. Additionally, the combination of GVAX, anti-PD1, and CD137 agonism is associated with moderate pathologic responses in 3/10 patients and improved disease-free survival, both notable outcomes for such a lethal disease.^23^ However, this study examined only a subset of patients from an already small clinical trial, preventing analyses comparing TME changes associated with differences in patient outcomes. We therefore note the need for further examination of these therapies in more patients to determine efficacy.

Despite the limited sample size, we once again find that a combination immunotherapy regimen in PDAC alters cytoskeletal and ECM-interacting components. The PDAC ECM is typically characterized by a thickened collagen rich stroma that acts to inhibit the migration of T cells into the TME and prevent their direct interaction with tumor cells. Our finding that patients receiving the combination of GVAX, anti-PD1, and CD137 agonist demonstrate higher percent collagen area in their tumor tissue overall and directly surrounding lymphoid aggregates than GVAX alone or in combination with anti-PD1 is therefore unexpected given the associated improvements clinically. We hypothesize that these changes in ECM from therapy may modulate T cell trafficking, and thereby alter the efficacy of the therapy. The mechanism by which anti-PD1 and anti-CD137 agonist treatment is associated with cytoskeletal and ECM modulation therefore represents an important mechanistic question warrant further study using preclinical models and multi-omic integration of spatially resolved transcriptomic, proteomic, and ECM profiling in human PDAC.

Overall, our human neoadjuvant and adjuvant platform trial provides the opportunity to rapidly assess PDAC TME changes associated with different immunotherapies. As a result, ongoing data analysis of each prior treatment arm provides the opportunity to incorporate additional TME modulating agents with the goal of identifying the most effective combinations that enhance antitumor immunity. Effective combinations that correlate with indicators of improved survival are chosen for further clinical development. Altogether we found that the addition of CD137 agonist to GVAX and anti-PD1 is associated cancer cells and CAFs activating TREM2 signaling in TAMs. Strategies to target these TAMs may therefore be important additions to combination immunotherapy in PDAC. This study provides a unique resource of scRNA-seq data to identify immunotherapy-induced molecular changes within individual cells directly in human tumors, that generates novel hypotheses suggestive of new treatment strategies for PDAC to inform investigation in future preclinical studies and platform combination immunotherapy clinical trials.

### Limitations of the study

A critical limitation to our study is the small sample size of our trial limits the statistical power of this analysis and precludes the investigation of the effect of sex on immunotherapy-induced immune cell states. Therefore, future trials with larger sample sizes are essential to translate our findings to generate biomarkers for immunotherapy treatment in pancreatic cancer, and preclinical studies are needed to uncover the mechanisms suggested by the hypotheses generated in this human dataset for translation to therapeutic combinations. Moreover, profiling studies of human tumors can yield only correlations to generate hypotheses about the mechanisms underlying therapeutic response and resistance in contrast to the mechanistic insights enabled by perturbation studies that can be achieved in *in vitro* and *in vivo* models. Nonetheless, the computational extensions to our ligand-receptor network inference method enables unique discovery of cell signaling changes resulting from therapy directly in human tumors. Moreover, the scRNA-seq data generated in this study provides a unique resource of data that can be used to directly query mechanisms of immunosuppression in human PDAC tumors and thus to benchmark the human-relevance of future preclinical studies of PDAC immunotherapy through cross-species data integration in future work.

## Resource availability

### Lead contact

Further information and requests for resources and reagents should be directed to and will be fulfilled by the Lead contact: Elana J. Fertig (ejfertig@jhmi.edu).

### Materials availability

This study did not generate new unique reagents.

### Data and code availability

• Data have been uploaded to NIH GEO and dbGAP. RNA counts and TCR contigs have been deposited at GEO and are publicly available as of the date of publication. FASTQ files have been deposited at dbGAP under controlled access and are available after a formal request to the NIH Data Access Committee. Accession numbers are listed in the key resources table.
• Code has been deposited at Zenodo and is publicly available as of the date of publication. The accession numbers are listed in the key resources table.

## Acknowledgments

Funding for this work was provided through NIH/NCI grants R01CA197296, P30CA006973, P50CA062924, P01CA247886, U01CA253403, and the Lustgarten Foundation. We thank members of the Sidney Kimmel Comprehensive Cancer Center Experimental and Computational Genomics Core, supported by NIH/NCI grant P30CA006973, for assistance with next generation sequencing. J.M.M.’s postdoctoral training program is supported in part by the American Association of Immunologists Intersect Fellowship, and by NIH training grant T32CA009110. J.T.M. is supported by the 10.13039/100000054National Cancer Institute of the NIH under award number F31CA284525. J.T.M.’s predoctoral training program is supported in part by 10.13039/100000002NIH training grant 5T32GM07814. D.R.B.’s postdoctoral training program is supported by 10.13039/100000002NIH training grant T32CA153952. The content of this research is solely the responsibility of the authors and does not necessarily represent the official views of the National Institutes of Health. The graphical abstract was made using Biorender.

## Author contributions

Conceptualization: J.M.M., J.T.M., J.A.T., E.S.C., L.V.D., M.L., L.T.K., W.J.H., E.M.J., L.Z., and E.J.F. Software: J.M.M., J.T.M., J.A.T., L.V.D., A.D., D.R.B., P.L., W.H., Y.X., J.H.E., and E.J.F. Data curation: J.M.M., J.T.M., J.A.T., L.V.D., M.L., D.N.S., Q.Z., H.W., L.T.K., L.L.E., B.F.G., W.J.H., J.G., R.W., S.Y., and R.A.A. Resources: E.S.C., D.N.S., L.T.K., W.J.H., G.M., S.M., J.W.Z., R.A.A., E.M.J., L.Z., and E.J.F. Formal analysis: J.M.M., J.T.M., J.A.T., L.V.D., A.D., M.L., D.N.S., E.D.M., D.R.B., Q.Z., H.W., L.L.E., B.F.G., A.V.F., W.J.H., S.J.L., R.Z., P.L., J.G., R.W., A.V., W.H., and Y.X. Supervision: E.S.C., H.W., L.T.K., W.J.H., J.W.Z., J.H.E., S.Y., R.A.A., E.M.J., L.Z., and E.J.F. Funding acquisition: W.J.H., E.M.J., L.Z., and E.J.F. Validation: J.M.M., J.T.M., J.A.T., D.R.B., and W.J.H. Investigation: J.M.M., J.T.M., J.A.T., E.S.C., L.V.D., A.D., M.L., D.N.S., E.D.M., D.R.B., A.V.F., W.J.H., S.J.L., G.M., S.M., and S.Y. Visualization: J.M.M., J.T.M., J.A.T., L.V.D., M.L., D.N.S., and D.R.B. Methodology: J.M.M., J.T.M., J.A.T., L.V.D., A.D., M.L., D.N.S., D.R.B., L.T.K., A.V.F., W.J.H., J.G., G.M., S.M., W.H., Y.X., J.H.E., and R.A.A. Writing original draft: J.M.M., J.T.M., J.A.T., E.S.C., L.V.D., D.N.S., D.R.B., L.T.K., W.J.H., Y.X., E.M.J., L.Z., and E.J.F. Writing – review and editing: J.M.M., J.T.M., J.A.T., E.S.C., L.V.D., A.D., M.L., D.N.S., E.D.M., D.R.B., Q.Z., H.W., L.T.K., L.L.E., B.F.G., A.V.F., W.J.H., S.J.L., R.Z., P.L., J.G., G.M., S.M., R.W., A.V., W.H., Y.X., J.W.Z., J.H.E., S.Y., R.A.A, E.M.J., L.Z., and E.J.F.. Project administration: E.M.J., L.Z., and E.J.F.

## Declaration of interests

L.Z. receives grant support from Bristol Myers Squibb, Merck, Astrazeneca, iTeos, Amgen, NovaRock, Inxmed, and Halozyme. L.Z. is a paid consultant/advisory Board Member at Biosion, Alphamab, NovaRock, Ambrx, Akrevia/Xilio, QED, Natera, Novagenesis, Snow Lake Capitals, BioArdis, Amberstone Biosciences, Tempus, Pfizer, Tavotek Lab, Clinical Trial Options, LLC, and Mingruizhiyao. L.Z. holds shares at Alphamab, Amberstone, and Mingruizhiyao. E.M.J. reports other support from Abmeta and Adventris, personal fees from Achilles, Dragonfly, Mestag, The Medical Home Group, and Surgtx, other support from Parker Institute, grants and other support from the Lustgarten Foundation, Genentech, BMS, and Break Through Cancer outside the submitted work. E.S.C. receives grant support from Affimed GmbH, NextCure, Pfizer, Haystack Oncology, Regeneron and is a consultant for Seres Therapeutics and SIRTex. R.A.A. receives grant support from Bristol-Meyer Squibb, RAPT Therapeutics. R.A.A. is a paid consultant for Bristol-Meyer Squibb, Merck, and Astrazeneca. S.Y. reports grants from NIH and Maryland Cigarette Restitution Fund during the conduct of the study, grants and personal fees from Cepheid, other support from Digital Harmonic, and grants from Janssen and Bristol Myers Squibb outside the submitted work. J.H.E. was previously a consultant and holds equity in Unity Biotechnology, Aegeria Soft Tissue and is an advisor for Tessera Therapeutics, HapInScience, Regenity, and Font Bio. W.J.H. has patent royalties from Rodeo/Amgen, grants from Sanofi and NeoTX, and speaking/travel honoraria from Exelixis and Standard BioTools. J.W.Z. reports grant funding from Genentech.

## STAR★Methods

### Key resources table

**Table:** 

REAGENT or RESOURCE	SOURCE	IDENTIFIER
**Chemicals, peptides, and recombinant proteins**		

RPMI-1640	Gibco	Cat# 11875093
Percoll Density Gradient Media	Cytiva	Cat# CE17089101
5′ DGE Library and Gel Bead Kit (v3.1)	10x Genomics	PN-1000780
Single Cell V(D)J Enrichment Kit Human T cell	10x Genomics	PN-1000252
Masson Trichrome Stain	Mercedes Scientific	TRG-1

**Critical commercial assays**		

Chromium Controller	10x Genomics	PN-120223; RRID: SCR_02537
ImmunoSeq	Adaptive Technologies	Human TCRB
DNeasy Blood and Tissue kit	Qiagen	Cat# 69504

**Deposited data**		

Tumor-infiltrating Leukocyte RNA counts and TCR contigs (NIH GEO)	This paper	GEO: GSE279781
Tumor-infiltrating Leukocyte RNA and TCR FASTQ files (dbGAP)	This paper	dbGAP: phs003002.v2.p1

**Software and algorithms**		

R analysis scripts	This paper	Zenodo: https://doi.org/10.5281/zenodo.14197949
CellRanger (6.1.1)	10x Genomics	https://www.10xgenomics.com/support/software/cell-ranger/latest
R (v4.2.0; v4.3.0)	R Core Team^76^	https://www.r-project.org
Seurat (v4.3.0)	Hao et al.^77^	https://cran.r-project.org/web/packages/Seurat/index.html
Monocle3 (v1.0.0)	Trapnell et al.^78^	https://github.com/cole-trapnell-lab/monocle3
ggplot2 (v3.4.1)	Wickham^79^	https://cran.r-project.org/web/packages/ggplot2/index.html
Matrix.utils (v0.9.8)	Varrichio^80^	https://cran.r-project.org/src/contrib/Archive/Matrix.utils/
SVA (v3.40.0)	Leek et al.^81^	https://bioconductor.org/packages/release/bioc/html/sva.html
DEseq2 (v1.32.0)	Love et al.^82^	https://bioconductor.org/packages/release/bioc/html/DESeq2.html
ashr (v2.2-54)	Stevens et al.^83^	https://cran.r-project.org/web/packages/ashr/index.html
fgsea (v1.18.0)	Korotkevich et al.^84^	https://bioconductor.org/packages/release/bioc/html/fgsea.html
MsigDB (v7.4.1)	Liberzon et al.^85^	https://www.gsea-msigdb.org/gsea/msigdb
HALO (v3.0.0)	Indica Software	https://indicalab.com/halo/
QuPath (v0.5.0)	Bankhead et al.^86^	https://github.com/qupath/qupath
lme4 (v1.1–35.3)	Bates et al.^87^	https://cran.r-project.org/web/packages/lme4/index.html
lmertest (v3.1-3)	Kuznetsova et al.^88^	https://cran.r-project.org/web/packages/lmerTest/index.html
Immunarch (v1.0.0)	Nazarov et al.^89^	https://cran.r-project.org/web/packages/immunarch/index.html
Modified FEST pipeline	Danilova et al.^34^	https://github.com/ldanilova/fest
scRepertoire (v1.3.5)	Borcherding et al.^90^	https://www.bioconductor.org/packages/release/bioc/html/scRepertoire.html
Domino (v0.1.1)	Cherry et al.^45^	Zenodo: https://zenodo.org/records/8277233
pySCENIC (v0.11.0)	Van de Sande et al.^91^	https://github.com/aertslab/pySCENIC
Circlize (v0.4.15)	Gu et al.^92^	https://cran.r-project.org/web/packages/circlize/index.html

### Experimental model and study participant details

#### Study participant details

**Table:** 

Patient	Arm	Age at Surgery	Sex	OS
P-45	Arm A	<65	F	>2 years
P-44	Arm A	≥65	F	<2 years
P-2	Arm A	<65	M	>2 years
P-42	Arm A	<65	F	>2 years
P-6	Arm A	<65	M	<2 years
P-20	Arm A	≥65	F	>2 years
P-33	Arm A	≥65	F	>2 years
P-24	Arm B	≥65	M	>2 years
P-32	Arm B	<65	M	>2 years
P-17	Arm B	<65	M	>2 years
P-3	Arm B	<65	M	>2 years
P-25	Arm C	≥65	F	>2 years
P-23	Arm C	<65	F	<2 years
P-46	Arm C	≥65	M	>2 years
P-40	Arm C	<65	F	>2 years

This study consists of clinical samples from 15 human subjects. 7 patients were assigned to Arm A, 4 patients were assigned to Arm B, and 4 patients were assigned to Arm C. Assignment of patients to treatment arms is described in detail in Heumann et al.^23^ In brief, patients were initially enrolled and randomized 1:1 between Arms A and B. When Arm C was added to the study, newly enrolled patients were consecutively enrolled in Arm C until the accrual goal was met due to discontinuation of urelumab production. Randomized enrollment into Arms A and B resumed until the completion of their accrual goals.

### Method details

#### Ethics and compliance

This study was approved by the Johns Hopkins Institutional Review Board (IRB) and Institutional Biosafety Committee (IBC) (Johns Hopkins Protocol #IRB00050517), the FDA Center for Biologics Evaluation and Research, and the National Institutes of Health Recombinant DNA Advisory Committee (ClinicalTrials.gov ID NCT02451982) as described in Heumann et al.^23^

##### Sample preparation and sequencing

Single cell suspension was obtained freshly from a non-necrotic tumor piece at approximately 2 cm in diameter dissected by a pathologist after specimens for clinical diagnosis were reserved. RPMI-1640 media were used to resuspend the cells after mechanical disruption and homogenization of the tumor tissues. The cell suspension was filtered by a 70μm cell strainer into a layer of mononuclear cells followed by Percoll (Cytiva) density gradient centrifugation. The single cell suspensions were washed three times using 40 mL of the complete RPMI media each time. After the final wash with the RPMI media, single cell suspensions were cryopreserved in the liquid nitrogen freezer. For single cell sequencing, the cryopreserved single cell suspensions were thawed at 37°C; and the final concentration was adjusted to 1000–2000 viable cells/μL. To achieve a target of 10,000 cells per sample, approximately 17,000 cells in reverse transcriptase enzyme-buffer mix were loaded onto the Chromium single cell controller (10x Genomics) to generate droplet emulsion of single-cells and barcoded gel beads, for single cell digital gene expression and paired TCR sequencing, by using the single cell 5′ DGE Library and Gel Bead Kit v3.1 (10x Genomics), Single Cell V(D)J Enrichment Kit (Human T cell) and Chromium Next GEM Chip G Single Cell Kit (10x Genomics) according to the manufacturer’s instructions. The libraries for digital gene expression were sequenced using NovaSeq 6000 system (Illumina, San Diego, CA) with targeted sequencing depth of 50,000 paired-end reads per cell. Full length V(D)J segments were enriched from cell barcoded cDNA, which allowed to match alpha and beta chains of TCR and whole transcriptome of T-cells. The V(D)J libraries for TCR sequencing were sequenced using the HiSeq X platform (Illumina, San Diego, CA) with targeted sequencing depth of 5,000 paired end reads per cell.

##### Single cell preprocessing

CellRanger outputs were loaded into R^76^ (v4.2.0) using Seurat^77^ (v4.3.0). A Seurat object was created using a minimum of 3 cells per feature, with a minimum of 200 features per cell as the initial filtering. Additional filtering was completed using less than 25% mitochondrial genes and between >200 & <2500 unique features per cell to remove low quality and empty droplets as well as any doublets that may have been sequenced. The Seurat object was converted to Monocle3 object for preprocessing and batch correction. Preprocessing was completed using Monocle3^78^ (v1.0.0) with 50 dimensions. Dimensions were further reduced using 25L neighbors and 2 components. Cells were clustered with a resolution of 1e-6, and batch correction was performed using BatchelorR built into the Monocle3 framework during the align_cds function. Annotation of cell types was completed using the markers present in Table S2. Plotting was performed with the ggplot2^79^ (v3.4.1) package.

##### Differential gene expression and gene set enrichment

Single cell expression data was pseudobulked based on patient and cell type by taking the sum of counts in the aggregate.Matrix function of Matrix.utils^80^ (v0.9.8). Pseudobulked samples next underwent quality control metrics to verify the upper quantile of counts were larger than zero for each sample. Within each cell type of interest, we used principal component analysis (PCA) of the variance stabilization transform (vst) RNA-seq data to evaluate sample clustering. To evaluate the batch effects present due to sequencing, we repeated PCA and identified that the samples were primarily clustered by sequencing batch. To remedy this visually, vst counts underwent batch correction using the ComBat function in SVA^81^ (v3.40.0). Differential expression analysis across treatment arms was completed for each cell type independently (CD8 T, TAM) with DESeq2^82^ (v1.32.0), including batch as a covariate in our model. Estimated fold changes were shrunk with ashr^83^ (v2.2-54) using lfcShrink to account for the variation in the samples. Genes were statistically significant if the absolute log2-fold changes after shrinkage were greater than 0.5 and the FDR-adjusted *p* value below 0.05. Gene set enrichment was run with fgsea^84^ (v1.18.0) using MSigDb^85^ (v7.4.1) pathways annotated in the HALLMARK, KEGG, REACTOME, GO, and NABA databases. Gene sets with FDR-adjusted *p* values below 0.05 were considered significantly enriched.

##### Trichome staining and analysis

Patient tissue sections were stained using Masson’s trichrome (Mercedes Scientific) and digitally scanned. Annotations of lymphoid aggregates (LAs) were performed using HALO artificial intelligence, with training data provided by an expert pathologist and output verified by the same pathologist. Annotations and images were analyzed with QuPath^86^ (v0.5.0 for MacOS Arm64). Regions of interest (ROIs) were used for color deconvolution. Pixels were thresholded on the channel corresponding to the methyl blue collagen stain, with those above 0.25 marked as positive for collagen. No smoothing was applied during thresholding. Every annotation of LA was expanded using the Dilate Annotation Plugin with radii of 10, 50, 100, 150, and 200 microns, excluding the original annotation (Figure S3). These expanded annotations were restricted to the tissue to avoid non-tissue regions. Using the color thresholding method, the percentage of collagen-positive pixels in each of these six regions (the original annotation and the five expansions) for a single annotation was measured and averaged. These values are reported as percent collagen area (CA). Additionally, we measured the CA over the entire tissue for each whole slide image. The images, annotations, classifiers, and scripts used are available in the Supplement.

We hypothesized that the treatment protocol would affect the percent CA in and around TLSs. We further assumed random effects by patient and by the region (inside the TLS, within 10 microns, within 50 microns, etc.). Therefore, we fit a linear mixed-effects model using the lme4^87^ R package (v1.1–35.3). Analysis of Variance (ANOVA) was employed to compare the fixed effects of the treatment arms on percent CA from this model. *p*-values were computed using the lmerTest^88^ R package (v3.1-3). The analysis was conducted in R (v4.4.0). The percent CA measurements and the script used to analyze them are available in the Supplement.

##### Bulk TCR-seq analysis

Bulk TCR beta chain sequencing was performed using Adaptive Biotechnologies immunoSEQ assay. Genomic DNA was purified from peripheral blood mononuclear cells (PBMCs) from all patients in our single-cell cohort at baseline (pre-treatment) and at time of surgical resection two weeks after treatment (post-treatment, matched timepoint to the scRNA-seq TILs) using the Qiagen DNeasy Blood and Tissue kit. The maximum amount of DNA (up to 20.6μg in 100μL) was sent to Adaptive Biotechnologies for sequencing. Resulting sequences were analyzed using Immunarch^89^ 1.0.0, and Fisher Tests were performed using a modified FEST pipeline (https://github.com/ldanilova/fest)^33^ using FDR adjusted *p*-value and odds ratio thresholds of <0.05 and >5, respectively, to classify clones as expanded or contracted. Clonality was calculated using normalized Shannon’s entropy (1- Shannon’s equitability).

##### TCR and differential expression for clonal T cells

TCR sequences were extracted using cellranger version 6.1.1 (VDJ reference vdj_GRCh38_alts_ensembl-5.0.0). Cellranger outputs were then loaded into R and combined with gene expression data using scRepertoire^90^ version 1.3.5 and Seurat version 4.3.0. Expansion UMAPs and barplots were generated using this integrated TCR and gene expression Seurat object. Cell cycle scores were calculated and cell cycle stages assigned using Seurat’s built-in CellCycleScoring function. Differential expression of expanded CD8^+^ T cells was performed using a Mast test^34^ with Seurat’s built-in FindMarkers function. Differentially expressed genes with a Bonferroni-adjusted *p* value below 0.05 were considered significant.

##### Differential cellular communication analysis

Inference of cellular communication interactions between cells in scRNA-seq data was carried out using Domino^44^ software (version 0.1.1). After quality control filtering and cell type annotation, cells in the TIL scRNA-seq data were divided into groups based on patients from which they originated. Subsequent analyses with Domino were carried out on a per-patient basis. Transcription factor activity scores were calculated using pySCENIC^91^ (version 0.11.0) with GRCh38/hg38 reference transcription factor binding sites. Identification of genes encoding interacting ligands and receptors was based on annotations in CellPhoneDB^93^ (version 2.0.0). Domino was used to identify active transcription factors in each cell type cluster based on significant differences in pySCENIC transcription factor activity scores by Wilcoxon test against other cell types (*p* < 0.001) and to identify active receptors instigating the transcription factor activity based on Spearman correlation of scaled expression of receptor expression with transcription factor activity scores greater than 0.25. Possible ligands instigating active receptor signaling were compiled based on annotation as interacting partners with receptors of interest annotated in CellPhoneDB. Expression of these ligands by cell types within the scRNA-seq data was displayed in circos plots (circlize^92^ v0.4.15) where the width of chords connecting ligands to their corresponding receptors is based on the mean normalized expression of the ligand within the displayed cell type.

In this study, we sought to extend Domino to compare changes in the ligand-receptor networks between treatment groups. For each patient, lists of active transcription factors, active receptors, and possible ligands called by Domino in each cell type were compiled for each patient. The quantity of active signal receipt by each cell type in each treatment arm was measured as the proportion of patients with active signal receipt in the cell type divided by the number of patients in the treatment arm. To test the dependence of receptor activity on treatment, the proportions of active signal receipt for a cell type were compared using Fisher’s exact test in the R stats package (version 4.2.0). Results were ordered by *p*-values derived from the Fisher’s exact test to prioritize receptors with the greatest degree of difference in activity across treatment arms, and *p*-values below 0.1 were prioritized as statistically significant.

Identification of possible ligands instigating active receptor signaling was carried out by comparison normalized expression of ligands across cell types to identify which ligand species was available in samples to instigate receptor activation. Identification of cell types as possible senders of ligands was carried out by differential expression using the MAST test in Seurat^77^ (version 4.1.1) to identify significant differences in ligand gene expression between cell types present in the scRNA-seq data.

### Quantification and statistical analysis

Differential gene expression was conducted with DESeq2^82^ (v1.32.0). *p*-values were FDR-adjusted based on the number of genes tested. Gene set enrichment analysis was conducted using Kolmagov-Smirnoff tests to calculate normalized enrichment score (NES) and *p*-value based on the test’s implementation in the fgsea R package^84^ (v1.18.0). *p*-values were FDR-adjusted based on the number of gene sets tested. Collagen area (CA) was compared between treatment groups by ANOVA to compare the fixed effects of the treatment arms on percent CA to distance from TLS. Differential Expression of genes between expanded T cell clones and of ligand-encoding genes between cell types were conducted by MAST test in Seurat.^77^ Testing for differential active receptors as inferred by Domino^44^ was conducted by Fisher’s Exact test.

### Additional resources

Additional details of the trial, data, contact information, proposal forms, and review and approval process are available at the following website: https://clinicaltrials.gov/ct2/show/NCT02451982.

## Supplemental information

Document S1. Figures S1–S6

Table S1. Marker genes for cell annotations

Table S2. CD8s differentially expressed genes

Table S3. Patients included in trichrome analyses

Table S4. Peripheral dynamics of hyperabundant and large tunor-infiltrating CD8^+^ T cell clones

Table S5. Patients included in abundant CD8^+^ T cell Differential Expression Analyses

Table S6. Clonal and abundant CD8s differentially expressed genes

Table S7. TAMs differentially expressed genes
