# Supplementary material for: CD137 agonism enhances anti-PD1 induced activation of expanded CD8+ T cell clones in a neoadjuvant pancreatic cancer clinical trial
Source: iScience. 2024 Dec 10;28(1):111569. doi: 10.1016/j.isci.2024.111569 (PMC11730579; doi:10.1016/j.isci.2024.111569)
Supplement: Document S1. Figures S1–S6 [file mmc1.pdf]

iScience, Volume *28*

Supplemental information

CD137 agonism enhances anti-PD1 induced activation of expanded CD8$^+$ T cell clones in a neoadjuvant pancreatic cancer clinical trial

Janelle M. Montagne, Jacob T. Mitchell, Joseph A. Tandurella, Eric S. Christenson, Ludmila V. Danilova, Atul Deshpande, Melanie Loth, Dimitrios N. Sidiropoulos, Emily Davis-Marcisak, Daniel R. Bergman, Qingfeng Zhu, Hao Wang, Luciane T. Kagohara, Logan L. Engle, Benjamin F. Green, Alexander V. Favorov, Won Jin Ho, Su Jin Lim, Rui Zhang, Pan Li, Jessica Gai, Guanglan Mo, Sarah Mitchell, Rulin Wang, Ajay Vaghasia, Wenpin Hou, Yao Xu, Jacquelyn W. Zimmerman, Jennifer H. Elisseeff, Srinivasan Yegnasubramanian, Robert A. Anders, Elizabeth M. Jaffee, Lei Zheng, and Elana J. Fertig

Figure S1

A

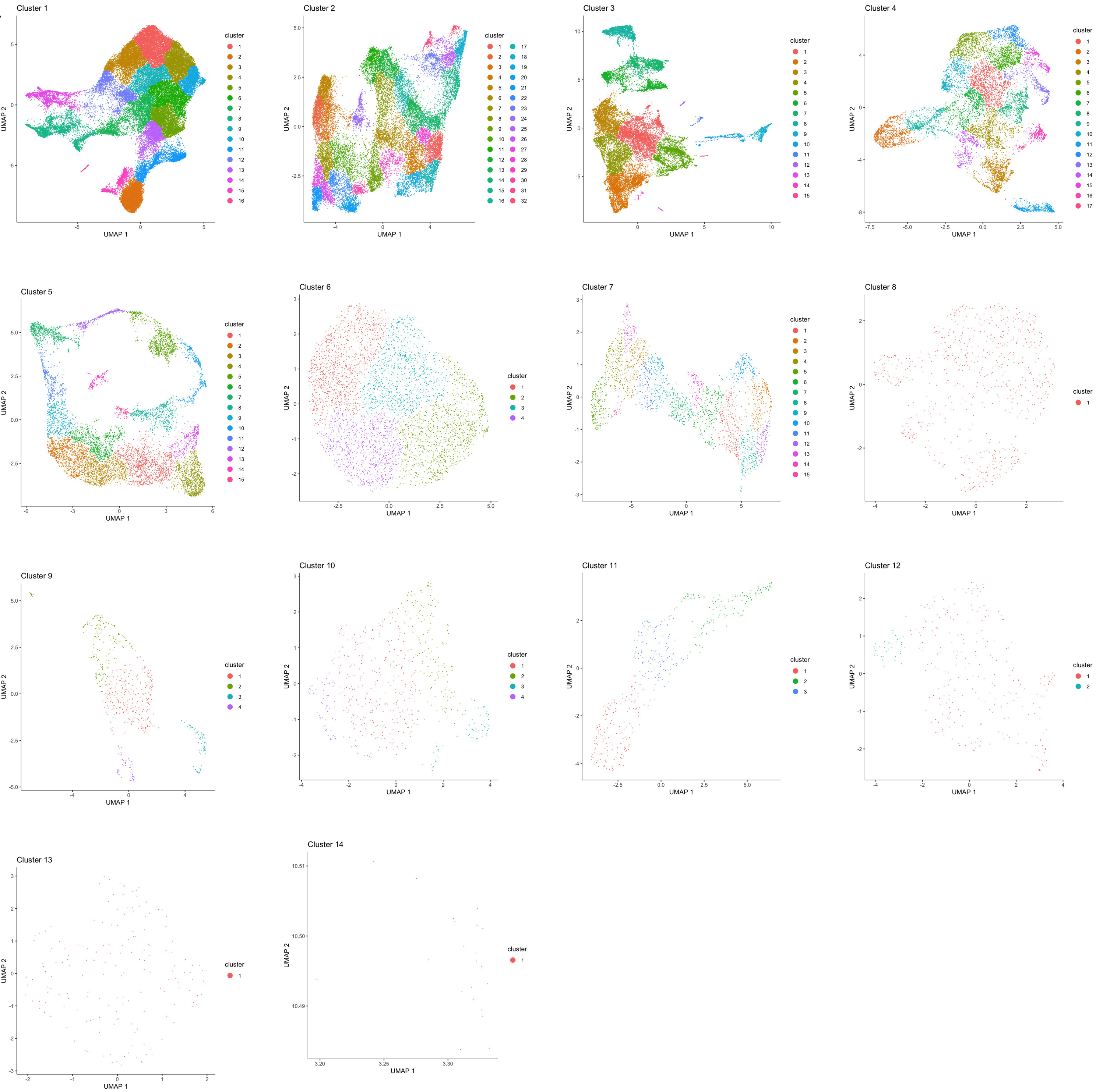
Cluster 1
Cluster 2
Cluster 3
Cluster 4
Cluster 5
Cluster 6
Cluster 7
Cluster 8
Cluster 9
Cluster 10
Cluster 11
Cluster 12
Cluster 13
Cluster 14
UMAP 1
UMAP 2
cluster

Figure S2

A

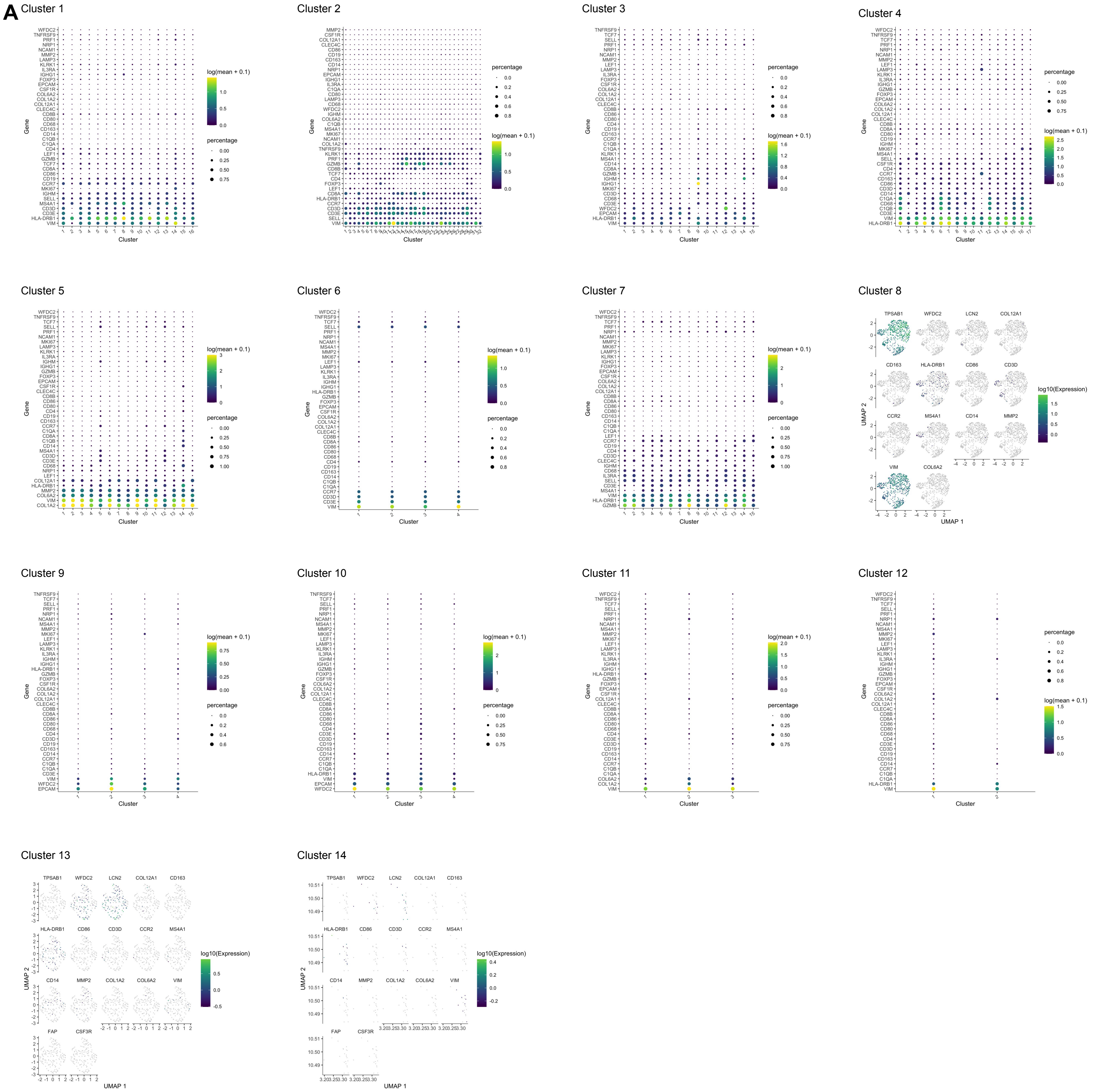
Cluster 1
Cluster 2
Cluster 3
Cluster 4
Cluster 5
Cluster 6
Cluster 7
Cluster 8
Cluster 9
Cluster 10
Cluster 11
Cluster 12
Cluster 13
Cluster 14
Gene
Cluster
log(mean + 0.1)
percentage
log10(Expression)
UMAP 1
UMAP 2
TPSAB1
WFDC2
LCN2
COL12A1
CD163
HLA-DRB1
CD86
CD3D
CCR2
MS4A1
CD14
MMP2
COL1A2
COL6A2
VIM
FAP
CSF3R

Figure S3

A

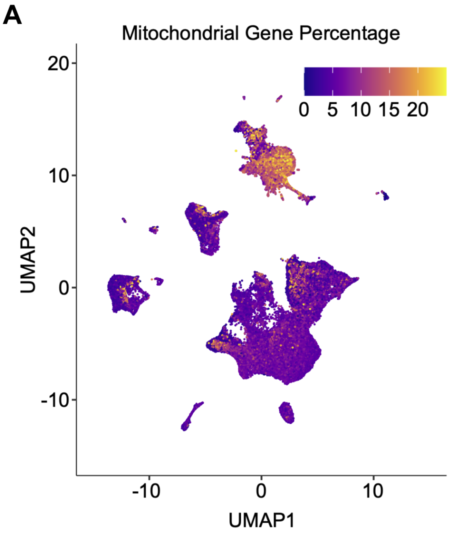

Mitochondrial Gene Percentage
0 5 10 15 20
20
10
0
-10
UMAP2
-10 0 10
UMAP1

Figure S4

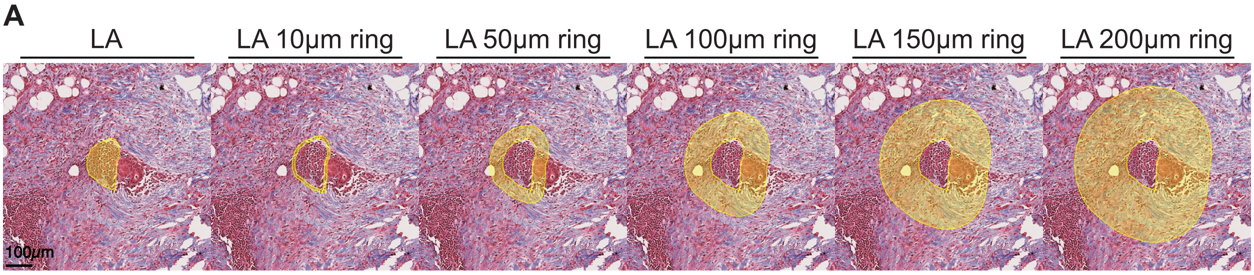
A
LA
LA 10μm ring
LA 50μm ring
LA 100μm ring
LA 150μm ring
LA 200μm ring
100μm

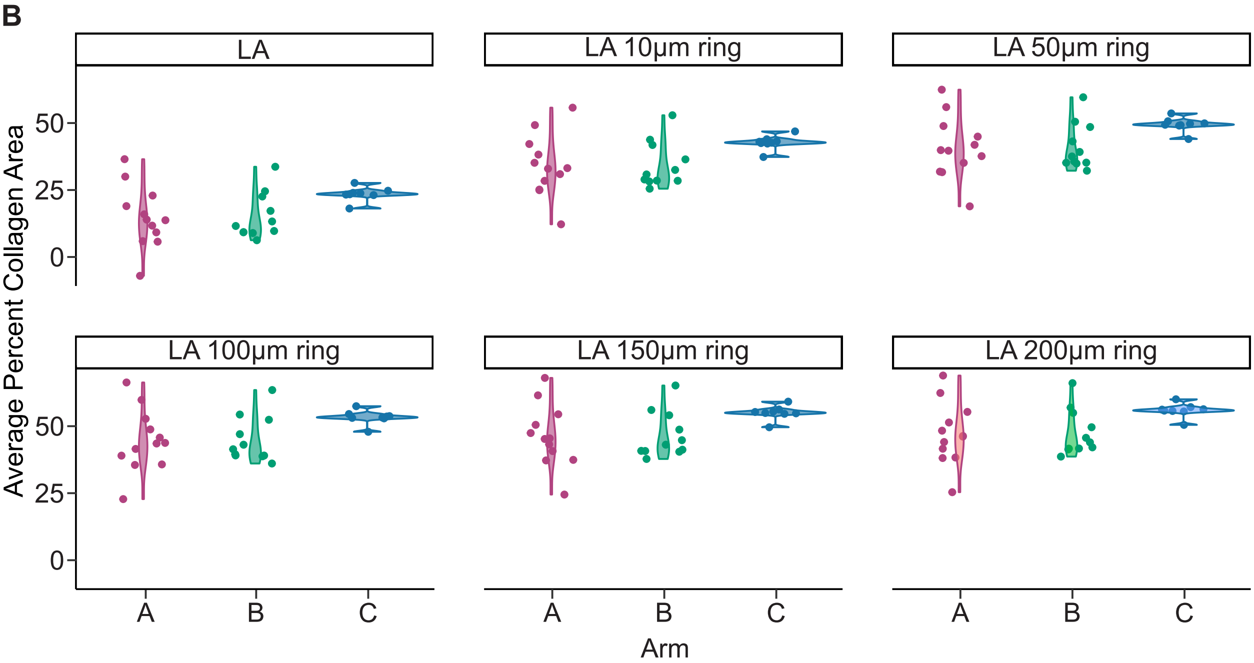
B
LA
LA 10μm ring
LA 50μm ring
LA 100μm ring
LA 150μm ring
LA 200μm ring
Average Percent Collagen Area
50
25
0
A
B
C
Arm

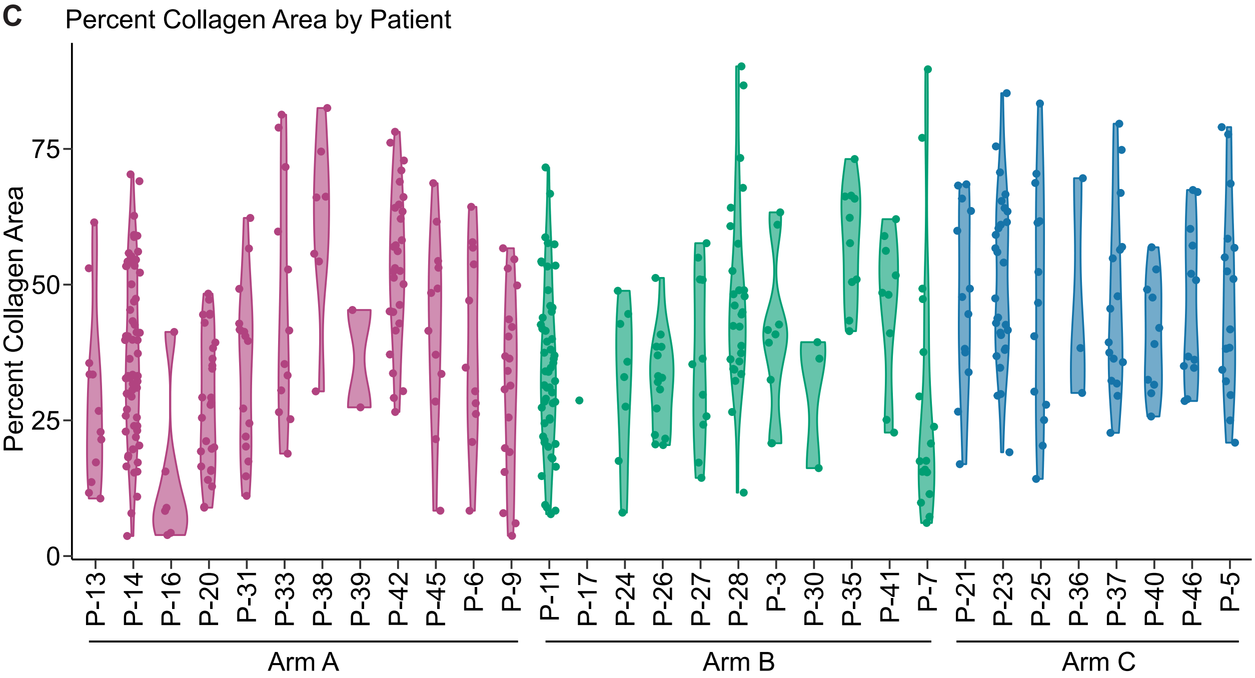
C
Percent Collagen Area by Patient
Percent Collagen Area
75
50
25
0
P-13
P-14
P-16
P-20
P-31
P-33
P-38
P-39
P-42
P-45
P-6
P-9
P-11
P-17
P-24
P-26
P-27
P-28
P-3
P-30
P-35
P-41
P-7
P-21
P-23
P-25
P-36
P-37
P-40
P-46
P-5
Arm A
Arm B
Arm C

Figure S5

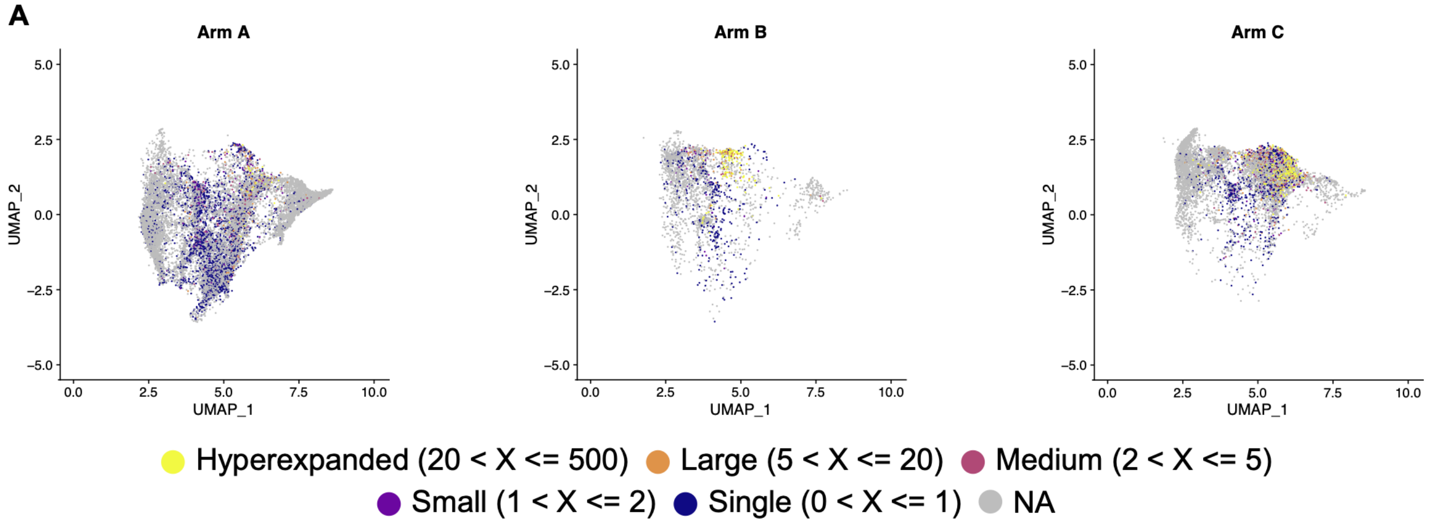
A
Arm A
Arm B
Arm C
UMAP_2
UMAP_1
5.0
2.5
0.0
−2.5
−5.0
0.0
2.5
5.0
7.5
10.0
Hyperexpanded (20 < X <= 500)
Large (5 < X <= 20)
Medium (2 < X <= 5)
Small (1 < X <= 2)
Single (0 < X <= 1)
NA

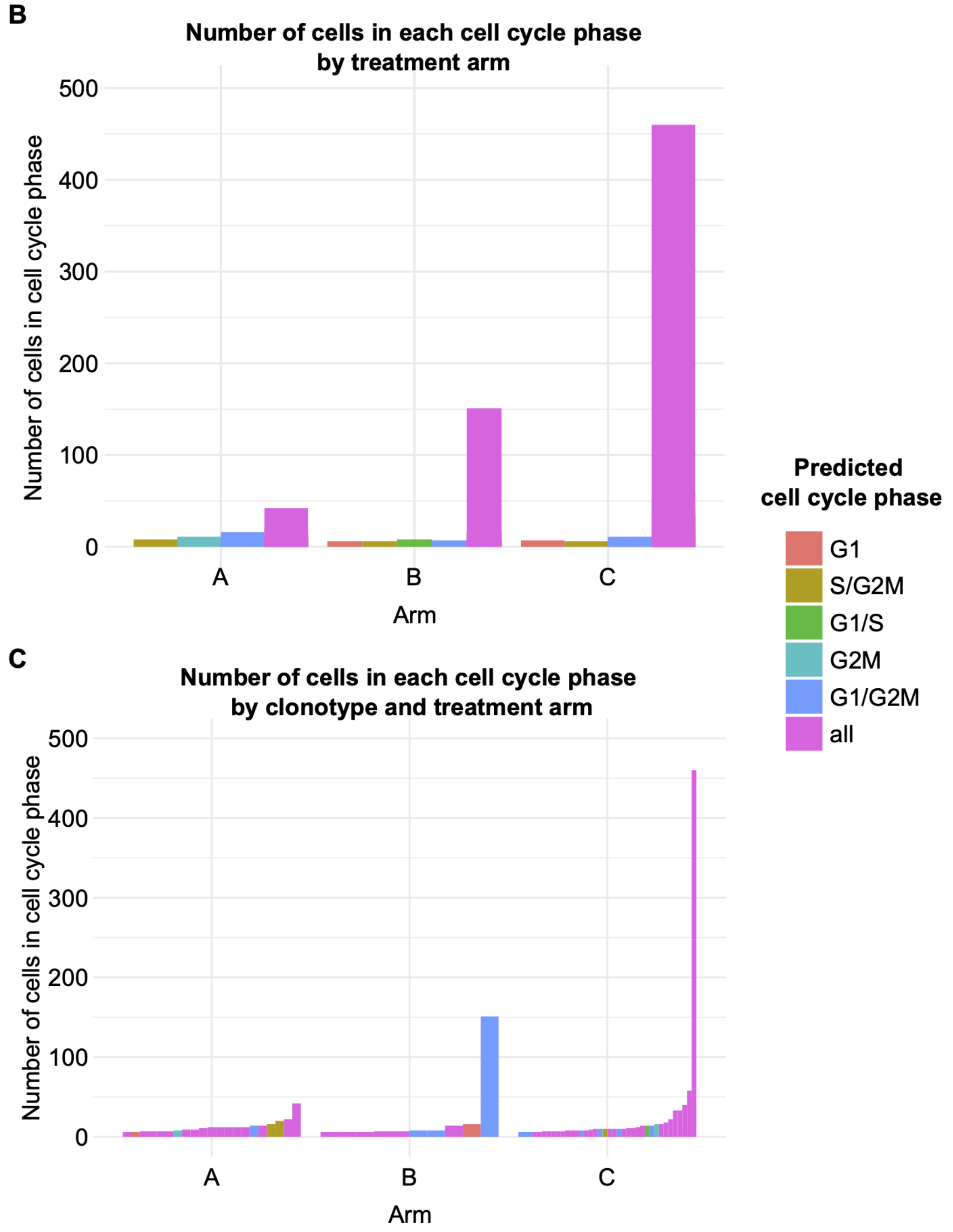
B
Number of cells in each cell cycle phase
by treatment arm
Number of cells in cell cycle phase
500
400
300
200
100
0
A
B
C
Arm
C
Number of cells in each cell cycle phase
by clonotype and treatment arm
Number of cells in cell cycle phase
500
400
300
200
100
0
A
B
C
Arm
Predicted
cell cycle phase
G1
S/G2M
G1/S
G2M
G1/G2M
all

Figure S6

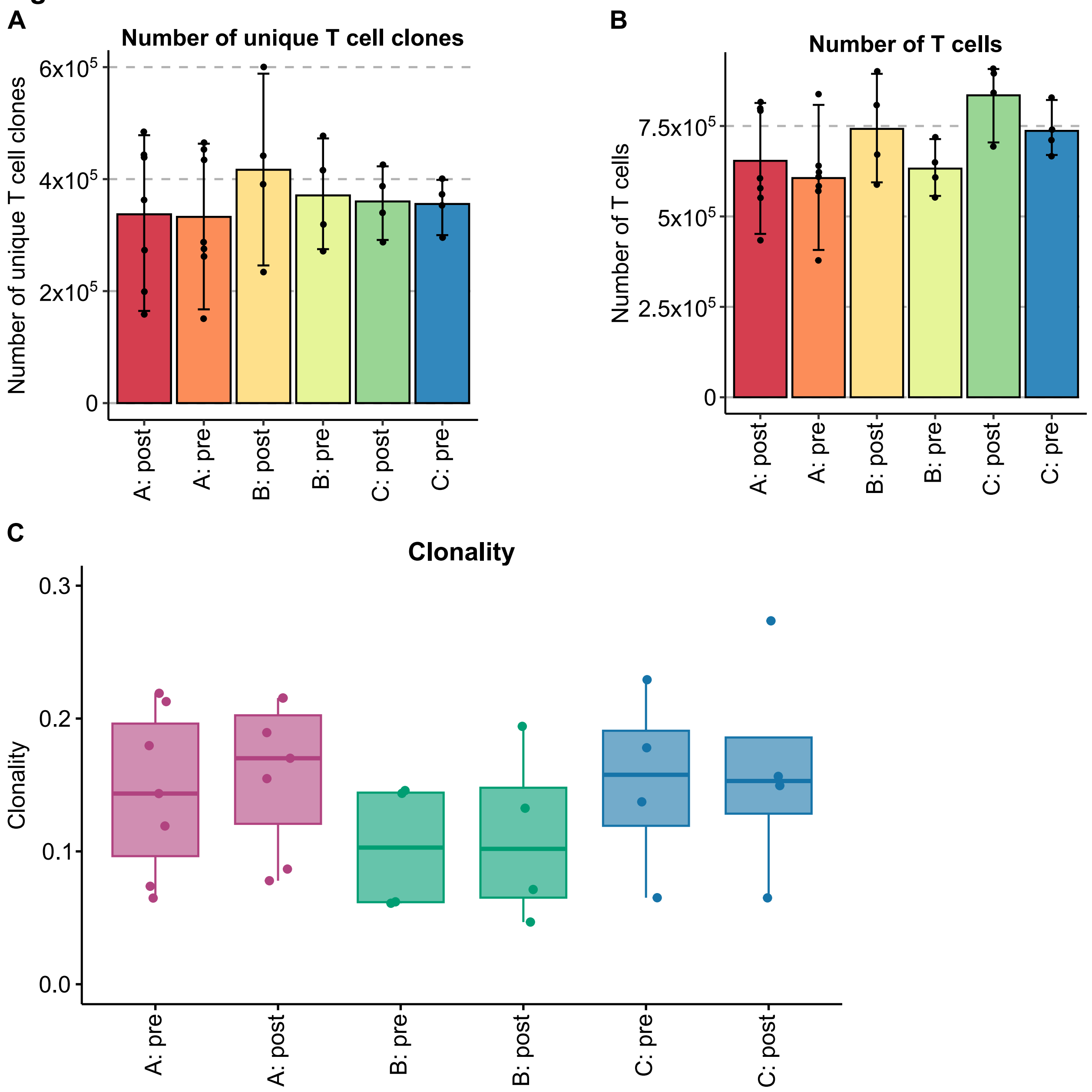
A
Number of unique T cell clones
Number of unique T cell clones
6x10^5
4x10^5
2x10^5
0
A: post
A: pre
B: post
B: pre
C: post
C: pre
B
Number of T cells
Number of T cells
7.5x10^5
5x10^5
2.5x10^5
0
A: post
A: pre
B: post
B: pre
C: post
C: pre
C
Clonality
Clonality
0.3
0.2
0.1
0.0
A: pre
A: post
B: pre
B: post
C: pre
C: post

Figure S1: Reclustered UMAPs used for cell annotation. A. Major clusters from Figure 1 were reclustered and explored for cell marker genes as shown in Figure S2.

Figure S2: Summary of marker genes and cell annotations by cluster. A. Marker genes used to annotate each cluster based on the reclustering shown in Figure S1. These annotations were applied to the cells in the original UMAP from Figure 1.

Figure S3: Mitochondrial Gene Expression. A. UMAP showing mitochondrial gene expressions levels across the entire UMAP.

Figure S4: Summary of Masson's trichrome staining on tissue sections from post-treatment tumors. A. Example LA and 10-200um rings used for analysis. **B.** Percent CA by arm for each region surrounding LAs in the dataset. C. Percent CA for each LA by patient, grouped by treatment arm.

Figure S5: Summary of scRNA-seq T cell clonal expansion by treatment arm. A. UMAPs by arm depicting T cell expansion status (all 3 overlaid in Figure 3B). **B.** Predicted cell cycle states for abundant and clonal CD8+ T cells by arm and **C.** by unique clonotype and arm.

Figure S6: Summary statistics of bulk TCR-seq data of pre- and post-treatment PBMCs. A. Number of unique T cell clones by arm and timepoint. **B.** Number of T cells by arm and timepoint. **C.** Clonality by arm and timepoint.
